# Acemetacin cocrystals and salts: structure solution from powder X-ray data and form selection of the piperazine salt

**DOI:** 10.1107/S2052252514004229

**Published:** 2014-02-28

**Authors:** Palash Sanphui, Geetha Bolla, Ashwini Nangia, Vladimir Chernyshev

**Affiliations:** a School of Chemistry, University of Hyderabad, Prof. C. R. Rao Road, Central University PO, Hyderabad 500046, India; b Department of Chemistry, M. V. Lomonosov Moscow State University, 1–3 Leninskie Gory, Moscow 119991, Russian Federation; c A. N. Frumkin Institute of Physical Chemistry and Electrochemistry RAS, 31 Leninsky Prospect, Moscow 119071, Russian Federation

**Keywords:** cocrystal, hydrate, melt crystallization, piperazine, powder X-ray diffraction, structure determination from powder data (SDPD)

## Abstract

Acemetacin (ACM) is a non-steroidal anti-inflammatory drug (NSAID), which causes reduced gastric damage compared with indomethacin. However, acemetacin has a tendency to form a less soluble hydrate in the aqueous medium. We noted difficulties in the preparation of cocrystals and salts of acemetacin by mechanochemical methods, because this drug tends to form a hydrate during any kind of solution-based processing. With the objective to discover a solid form of acemetacin that is stable in the aqueous medium, binary adducts were prepared by the melt method to avoid hydration. The coformers/salt formers reported are pyridine carboxamides [nicotinamide (NAM), isonicotinamide (INA), and picolinamide (PAM)], caprolactam (CPR), *p*-aminobenzoic acid (PABA), and piperazine (PPZ). The structures of an ACM–INA cocrystal and a binary adduct ACM–PABA were solved using single-crystal X-ray diffraction. Other ACM cocrystals, ACM–PAM and ACM–CPR, and the piperazine salt ACM–PPZ were solved from high-resolution powder X-ray diffraction data. The ACM–INA cocrystal is sustained by the acid⋯pyridine heterosynthon and N—H⋯O catemer hydrogen bonds involving the amide group. The acid⋯amide heterosynthon is present in the ACM–PAM cocrystal, while ACM–CPR contains carboxamide dimers of caprolactam along with acid–carbonyl (ACM) hydrogen bonds. The cocrystals ACM–INA, ACM–PAM and ACM–CPR are three-dimensional isostructural. The carboxyl⋯carboxyl synthon in ACM–PABA posed difficulty in assigning the position of the H atom, which may indicate proton disorder. In terms of stability, the salts were found to be relatively stable in pH 7 buffer medium over 24 h, but the cocrystals dissociated to give ACM hydrate during the same time period. The ACM–PPZ salt and ACM–nicotinamide cocrystal dissolve five times faster than the stable hydrate form, whereas the ACM–PABA adduct has 2.5 times faster dissolution rate. The pharmaceutically acceptable piperazine salt of acemetacin exhibits superior stability, faster dissolution rate and is able to overcome the hydration tendency of the reference drug.

## Abstract 2

Multi-component crystals of the anti-inflammatory drug acemetacin were prepared by melt crystallization and their X-ray crystal structures solved using single-crystal and high-resolution powder X-ray diffraction (PXRD) data. The acemetacin–*para*-aminobenzoic acid adduct and the acemetacin piperazine salt are stable to hydration in the aqueous medium (up to 24 h).

## 1. Introduction   

Combinatorial chemistry and high-throughput screening of drug molecules (Lipinski *et al.*, 1997 ▶, 2012 ▶; Homon & Nelson, 2006 ▶) can yield new hits and leads in medicinal chemistry programs, but at the same time the drug industry has to cope with poor aqueous solubility, stability and low bioavailability at the pharmaceutics stage of drug development. Active pharmaceutical ingredients (APIs) may exist as single-component (polymorphs, amorphous phases) or multi-component systems (cocrystals, salts, eutectics, solvates, hydrates; Morissette *et al.*, 2004 ▶; Mukherjee *et al.*, 2011 ▶; Rajput *et al.*, 2013 ▶). To achieve optimal aqueous solubility of the drug, methods such as salt formation, micronization, emulsification, use of surfactants, solid dispersions, polymeric drug carriers or cyclodextrin complexes (Serajuddin, 2007 ▶; Sharma *et al.*, 2009 ▶; Anand *et al.*, 2007 ▶) have been used. The efficacy of these methods depends on the physicochemical nature of the drug molecule, and there is no universal remedy which can satisfactorily solve all issues. Even so, salts are generally most preferred in the pharmaceutical industry because of their high solubility, improved stability, better crystallinity, filterability and manufacturing processes (Braga *et al.*, 2013 ▶; Sanphui *et al.*, 2012 ▶; Bhatt *et al.*, 2005 ▶). On the down side, however, salts being ionic in nature tend to be more hygroscopic than neutral cocrystals, and this single fact can sometimes counter the above-mentioned advantages. Cocrystals have been modified to have improved tableting properties (Karki *et al.*, 2009 ▶), as high-energy materials (Landenberger *et al.*, 2013 ▶), optical materials (D’Silva *et al.*, 2011 ▶), and to control the chemical degradation of drugs (Babu *et al.*, 2012 ▶). Cocrystals can be engineered to tune solubility, bioavailability and stability of the drug (Remenar *et al.*, 2003 ▶; McNamara *et al.*, 2006 ▶; Smith *et al.*, 2013 ▶). The US-FDA recently defined pharmaceutical cocrystals in terms of acid–base adducts in which Δp*K*_a_ [(conjugate acid of base) − p*K*_a_ (acid)] < 1 and stated that the API must dissociate from the cocrystal before reaching the target receptor site (US-FDA, 2013*a* ▶). If Δp*K*_a_ > 1, salt formation is likely and this must be confirmed by spectroscopic and/or diffraction techniques. It has been noted that the region 1 < Δp*K*_a_ < 3 is a grey zone in which cocrystal, salt or a salt–cocrystal continuum may exist (Childs *et al.*, 2007 ▶; Sarma *et al.*, 2009 ▶; Paluch *et al.*, 2011 ▶).

Acemetacin (ACM; Chávez-Piña *et al.*, 2007 ▶) is a glycolic acid ester prodrug of indomethacin. The main advantage with acemetacin is that it moderates gastric acidity effects associated with indomethacin. The bioavailability of indomethacin after oral administration of its prodrug acemetacin is significantly reduced by acute hepatitis (Dell *et al.*, 1980 ▶; Chávez-Piňa *et al.*, 2009 ▶). Non-steroidal anti-inflammatory drugs (NSAIDs) are used in the treatment of osteoarthritis, rheumatoid arthritis, lower back pain and post-operative inflammation by inhibiting prostaglandin synthesis. Acemetacin is sold under the trade name *Emflex* as 60 mg capsules (highest dose 180 mg). It is metabolised to indomethacin, which then acts as an inhibitor of cyclooxygenase to produce the anti-inflammatory effects. According to the British Pharmacopeia (2009 ▶), ACM is practically insoluble in water at acidic pH, whereas it is soluble in acetone and slightly soluble in anhydrous ethanol. Castro *et al.* (2001 ▶) reported that the solubility of ACM, which is a carboxylic acid drug, rapidly decreases in acidic media from 1.95 g L^−1^ at pH 7.4 to 23 mg L^−1^ at pH 5. We observed that acemetacin transforms to a monohydrate (Burger & Lettenbichler, 1993 ▶; Gelbrich *et al.*, 2007 ▶) during crystallization and grinding in ordinary solvents, possibly due to the polar glycolic ester group, whereas indomethacin has no hydration issues post processing and administration. Trask *et al.* (2005 ▶, 2006 ▶) reported an elegant solution to the hydration problem of caffeine and theophylline in its cocrystal with oxalic acid. A solid form of acemetacin with good hydrolytic stability may overcome the above-mentioned problems. With this background, we started this study to improve the hydrolytic stability of acemetacin by screening cocrystals and salts for optimal form selection. In continuation of our preliminary report (Sanphui *et al.*, 2013 ▶) on acemetacin polymorphs (Yoneda *et al.*, 1981 ▶; Burger & Lettenbichler, 1993 ▶), we now discuss cocrystals and salts of ACM with pharmaceutically acceptable molecules (chemicals from the GRAS list; US-FDA, 2013*b* ▶), such as nicotinamide (NAM), isonicotinamide (INA), picolinamide (PAM), caprolactam (CPR), *p*-aminobenzoic acid (PABA) and piperazine (PPZ). The idea was to optimize strong heterosythons (Desiraju, 1995 ▶; Shattock *et al.*, 2008 ▶; Babu *et al.*, 2007 ▶) of the carboxylic acid group in ACM with partner molecules, so that the enthalpy gain through hydrogen bonding will minimize hydration of the drug cocrystal.
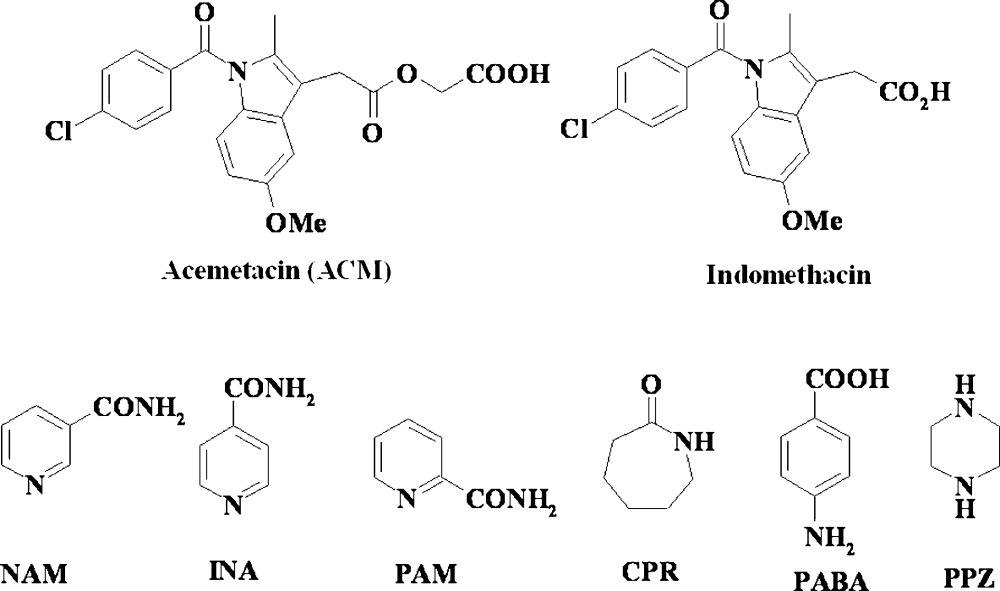


## 2. Experimental   

### 2.1. Materials   

Acemetacin was purchased from Dalian Hong Ri DongSheng Import & Export Co. Ltd, China (http://dlhongridongsheng.guidechem.com/) and used without further purification for all experiments. The commercial sample was confirmed to be stable form II of acemetacin (Sanphui *et al.*, 2013 ▶). All coformers were obtained from Sigma–Aldrich (Hyderabad, India) and solvents for crystallizations were of analytical grade. Melting points were measured on a Fisher–Johns melting point apparatus. Water filtered through a double deionized purification system (AquaDM, Bhanu, Hyderabad, India) was used in all experiments.

### 2.2. Preparation of acemetacin cocrystal/salts   

Acemetacin and the coformer were ground together in a 1:1 stoichiometric ratio, kept in a 10 ml sample vial and melted at 160°C (except PABA at 190°C). The solid product was kept for crystallization in different organic solvents. ACM–INA cocrystal and ACM–PABA adduct were obtained from dry EtOAc solvent. ACM–NAM, ACM–PAM and ACM–CPR cocrystals and ACM–PPZ salt were prepared by melt crystallization only; mechanochemical grinding in a ball mill or slurry grinding and crystallization resulted in either physical mixtures or acemetacin hydrate. Crystallization by cooling of the melt gave a glassy phase which was crystallized from isobutyl methyl ketone. The products were confirmed to be homogeneous single phases by differential scanning calorimetry (DSC).

### 2.3. Crystal structures from single-crystal X-ray diffraction   

Single crystals of the ACM–INA cocrystal and ACM–PABA adduct were analyzed on an Oxford Diffraction Gemini/EOS CCD instrument (Oxford Diffraction, Yarnton, Oxford, England) equipped with Mo *K*α radiation. Data reduction was performed using *CrysAlis PRO* (Oxford Diffraction, 2008 ▶), and the crystal structures were solved and refined using *Olex2* (Dolomanov *et al.*, 2009 ▶) with anisotropic displacement parameters for non-H atoms. H atoms were experimentally located through Fourier difference electron-density maps, except for one H atom in ACM–PABA (as discussed in §3). All C—H atoms were geometrically fixed and refined as riding atoms. *X-Seed* (Barbour, 2001 ▶) was used to prepare the figures and packing diagrams.

### 2.4. Crystal structures from powder X-ray diffraction   

X-ray powder diffraction data for ground samples of ACM–PAM, ACM–CPR and ACM–PPZ were collected at room temperature (25°C) on a Panalytical EMPYREAN instrument with a linear X’celerator detector and non-monochromated Cu *K*α radiation (λ = 1.5418 Å). The unit-cell dimensions were determined using three indexing programs: *TREOR90* (Werner *et al.*, 1985 ▶), *ITO* (Visser, 1969 ▶) and *AUTOX* (Zlokazov, 1992 ▶, 1995 ▶). Based on systematic extinctions, the space group for ACM–PAM was determined as *P*2_1_. For ACM–CPR and ACM–PPZ, space group 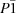 was chosen. The unit-cell parameters and space groups were further tested using a Pawley fit (Pawley, 1981 ▶) and confirmed by successful crystal structure solution and refinement. The crystal structures were solved by a simulated annealing technique (Zhukov *et al.*, 2001 ▶), taking into account the empirical formula, unit-cell volume and space-group symmetry. In the case of ACM–PPZ, these considerations led to the conclusion that the piperazine molecule must reside on an inversion centre. In simulated annealing runs, the ACM molecule required variations of 13 degrees of freedom: three positional, three translational and seven torsion parameters. The PAM, CPR and PPZ molecules were treated as rigid fragments, so that PPZ required only three orientational parameters (being fixed at the inversion centre) and PAM and CPR required six parameters (three translational and three orientational). The solutions found were fitted with the program *MRIA* (Zlokazov & Chernyshev, 1992 ▶) by bond-restrained Rietveld refinement using a split-type pseudo-Voigt peak-profile function (Toraya, 1986 ▶). Symmetrized harmonics expansion up to the fourth order (Ahtee *et al.*, 1989 ▶; Järvinen, 1993 ▶) was used for correction of any preferred orientation (texture) effect. Restraints were applied to the intramolecular bond lengths and contacts in all molecules, with the strength of the restraints applied as a function of interatomic separation and, for intramolecular bond lengths, corresponding to an r.m.s. deviation of 0.01 Å. Additional restraints were applied to the planarity of the rings and the attached atoms, with a maximum deviation allowed from the mean plane of 0.03 Å. All non-H atoms in ACM–PPZ were refined isotropically, while in ACM–PAM two common *U*_iso_ parameters were refined for non-H atoms in the ACM and PAM molecules, respectively. H atoms were positioned geometrically (C—H = 0.93–0.97, N—H = 0.86–0.90 Å) and not refined.

### 2.5. Powder X-ray diffraction   

Bulk samples were analyzed by powder X-ray diffraction using a Bruker AXS D8 powder diffractometer (Bruker AXS, Karlsruhe, Germany). Experimental conditions: Cu *K*α radiation (λ = 1.5406 Å); 40 kV, 30 mA; scan range 5–50° 2θ at a scan rate of 1° min^−1^; time per step 0.5 s. The experimental PXRD patterns and those calculated from the crystal structures were compared to confirm the purity of the bulk phase using *PowderCell* (Kraus & Nolze, 2000 ▶).

### 2.6. FT–IR spectroscopy   

IR spectra were recorded on samples dispersed in KBr pellets using a Thermo-Nicolet 6700 FT–IR spectrometer (Waltham, MA, USA).

### 2.7. Thermal analysis   

Differential scanning calorimetry (DSC) was performed on a Mettler Toledo DSC 822e module. Samples were placed in crimped but vented aluminium sample pans, with a typical sample size of 2–6 mg. The temperature range was 30–200°C at a heating rate of 5°C min^−1^. Samples were purged with a stream of dry N_2_ flowing at 150 ml min^−1^.

### 2.8. Solid-state NMR spectroscopy   

Solid-state (SS) ^13^C NMR spectra were obtained on a Bruker Ultrashield 400 spectrometer (Bruker BioSpin, Karlsruhe, Germany) utilizing a ^13^C resonant frequency of 100 MHz (magnetic field strength of 9.39 T). Approximately 100 mg of a fine crystalline sample was tightly packed into a zirconia rotor with the help of Teflon stick up to the cap Kel-F mark. A cross-polarization magic angle spinning (CP–MAS) pulse sequence was used for spectral acquisition. Each sample was spun at a frequency of 5.0 ± 0.01 kHz and the magic angle setting was calibrated by the KBr method. Each data set was subjected to a 5.0 Hz line-broadening factor and subsequently Fourier transformed and phase corrected to produce a frequency domain spectrum. The chemical shifts were referenced to TMS using glycine (δ_glycine_ = 43.3 p.p.m.) as an external secondary standard. ^15^N CP–MAS spectra recorded at 40 MHz were referenced to glycine N and then the chemical shifts were recalculated to nitromethane (δ_glycine_ = −347.6 p.p.m.).

### 2.9. Scanning electron microscopy (SEM)   

The particle size and morphology of the acemetacin binary systems were examined with a Philips XL30 ESEM scanning electron microscope (SEM) using a beam voltage of 20 kV. Prior to SEM imaging, an ultra-thin layer of gold was coated using Quorum Fine coat Ion Sputter Q150R ES (operating at 10 mA for 3 min), in order to enhance the conductivity of the samples. The ground particles were dispersed on a carbon-coated copper grid.

### 2.10. Dissolution experiments   

Intrinsic dissolution rate (IDR) experiments were carried out on a USP-certified Electrolab TDT-08 L Dissolution Tester (Mumbai, India). A calibration curve was obtained for ACM and all binary systems by plotting an absorbance *versus* concentration curve obtained from the five known concentration solutions in pH 7 phosphate buffer medium. The slope of the plot gave the molar extinction coefficient (∊) using the Beer–Lambert law. Equilibrium solubility was determined in the same medium using the shake-flask method (Glomme *et al.*, 2005 ▶). To obtain the equilibrium solubility, 100 mg of each solid form was stirred for 24 h in 5 ml buffer at 37°C, and the corresponding absorbance was measured at 318 nm. The concentration of the saturated solution was calculated at 24 h, which is the equilibrium solubility of that form. There was no interference of absorbance by the aromatic coformers (used here) in the 318 nm region.

For the IDR experiments, 100 mg of each material was taken in the intrinsic attachment and compressed to a 0.5 cm^2^ pellet using a hydraulic press at a pressure of 2.5 ton inch^−2^ for 2 min. The pellet was compressed to provide a flat surface at one end and the other end was sealed. The pellet was then dipped into 900 ml pH 7 phosphate buffer medium at 37°C with the paddle rotating at 150 r.p.m. At regular intervals of 5–10 min, 5 ml of the dissolution medium was withdrawn and replaced by an equal volume of fresh medium to maintain a constant volume. Samples were filtered through 0.2 µm nylon filter and assayed for drug content spectrophotometrically at 318 nm. The amount of drug dissolved in each time interval was calculated using the calibration curve. The dissolution rates of the solid forms were computed from their IDR values.

## 3. Results and discussion   

New cocrystals of ACM with NAM, INA, PAM, CPR, an adduct with PABA, and a salt with PPZ were prepared by melt crystallization (Porter *et al.*, 2008 ▶; Seefeldt *et al.*, 2007 ▶; Berry *et al.*, 2008 ▶) as detailed in §2. Our initial experiments were selected based on mechanochemical methods, such as liquid assisted grinding (LAG) and neat grinding. However, ACM hydrate (ACMH; Burger & Lettenbichler, 1993 ▶; Gelbrich *et al.*, 2007 ▶) was observed as a side product in a majority of the LAG and neat grinding results. The new binary systems were characterized by FT–IR, DSC, PXRD, SS NMR and SEM. Among the six multi-component systems reported herein, single crystals were obtained for ACM–INA (1:1) and ACM–PABA (1:1), whereas the remaining cases gave microcrystalline powders. The crystal structures of ACM–PAM (1:1), ACM–CPR (1:1) cocrystals and ACM–PPZ (1:0.5) salt were solved from high-resolution powder X-ray diffraction data. This can be corroborated by advanced techniques used for the crystal structure determination of cocrystals/salts and polymorphs (Braga *et al.*, 2012 ▶; Ueto *et al.*, 2012 ▶; Chernyshev, Petkune *et al.*, 2013 ▶; Chernyshev, Shkavrov *et al.*, 2013 ▶). Optimization of the molecular geometry reduces the parameters necessary to model the structure from three per atom to about six per molecule (three for position and three for orientation of the molecule) plus any torsion angles for bonds that allow rotations. However, the final agreement of the structure determination from powder XRD (SDPD) with three-dimensional coordinates is non-trivial and each structure poses its own unique problems. Generally, SDPD structure solution provides less information (lower precision in bond lengths and angles) than single-crystal X-ray data due to the overlap of Bragg reflections (Lapidus *et al.*, 2010 ▶). Good agreement between the experimental and calculated X-ray diffraction pattern in the final Rietveld refinement confirmed the accuracy of the crystal structures.^1^ We were unable to obtain the structure of ACM–NAM by SDPD and the diffraction pattern only is reported as the signature pattern; no molecular level details are available at the present time. Crystallographic information is summarized in Table 1 ▶. The hydrogen-bonding synthons in the cocrystal/salt structures can be compared with the dimer/catemer structures of the ACM polymorphs (Sanphui *et al.*, 2013 ▶).

### 3.1. Acemetacin–isonicotinamide cocrystal (ACM–INA, 1:1)   

**Figure 1:**
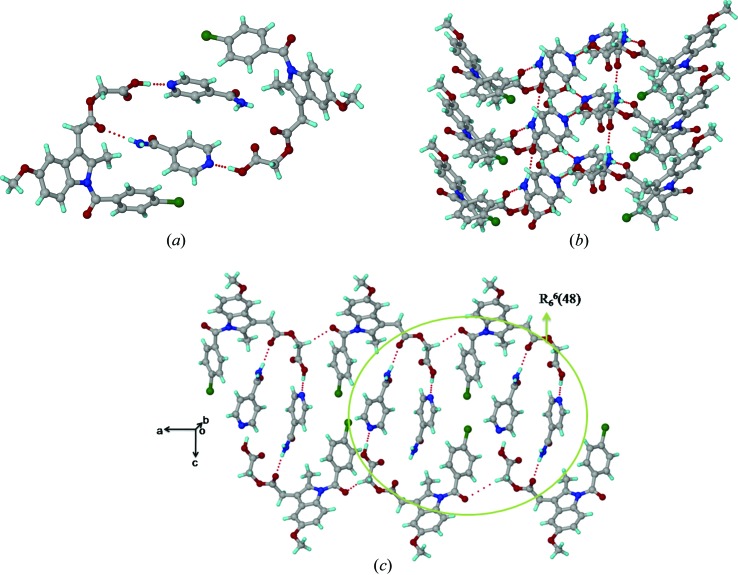
(*a*) O—H⋯N and N—H⋯O hydrogen bonds between ACM and INA. (*b*) INA molecules form an amide catemer chain through their *anti* N—H bonds. (*c*) *R*_6_^6^(48) ring motif in the ACM–INA cocrystal.

The asymmetric unit of the ACM–INA cocrystal (*P*2_1_, *Z* = 2) comprises one ACM and one INA molecule. INA interacts with two ACM molecules through the acid⋯pyridine heterosynthon *via* O—H⋯N (O6—H6*A*⋯N3; Table 2 ▶) and N—H⋯O hydrogen bonds (N2—H2*A*⋯O3; Table 2 ▶) between the *syn* N—H of INA and the ester carbonyl of ACM (Fig. 1 ▶ *a*). The *anti* N—H of the INA amide forms a catemer chain [graph set *C*(4); Etter *et al.*, 1990 ▶; Bernstein *et al.*, 1995 ▶] of N—H⋯O hydrogen bonds (N2—H2*B*⋯O7; Table 2 ▶) along the *b*-axis (Fig. 1 ▶ *b*). An N—H⋯O chain is present in all metastable forms of pure isonicotinamide (CSD refcodes EHOWIH02-05; Aakeröy *et al.*, 2003 ▶; Li *et al.*, 2011 ▶; Eccles *et al.*, 2011 ▶), while the carboxamide dimer is observed in the stable form (CSD refcode EHOWIH01; Aakeröy *et al.*, 2003 ▶). There is no acid⋯amide heterosynthon between ACM and the coformer. The amide carbonyl of ACM participates in auxiliary C—H⋯O interactions, forming one-dimensional chains parallel to the *a*-axis. Six INA molecules are sandwiched between six ACMs in the extended packing arrangement (Fig. 1 ▶ *c*). Four ACM and two INA molecules form a *R*_6_^6^(48) ring motif.

### 3.2. Acemetacin-picolinamide cocrystal (ACM–PAM, 1:1)   

**Figure 2:**
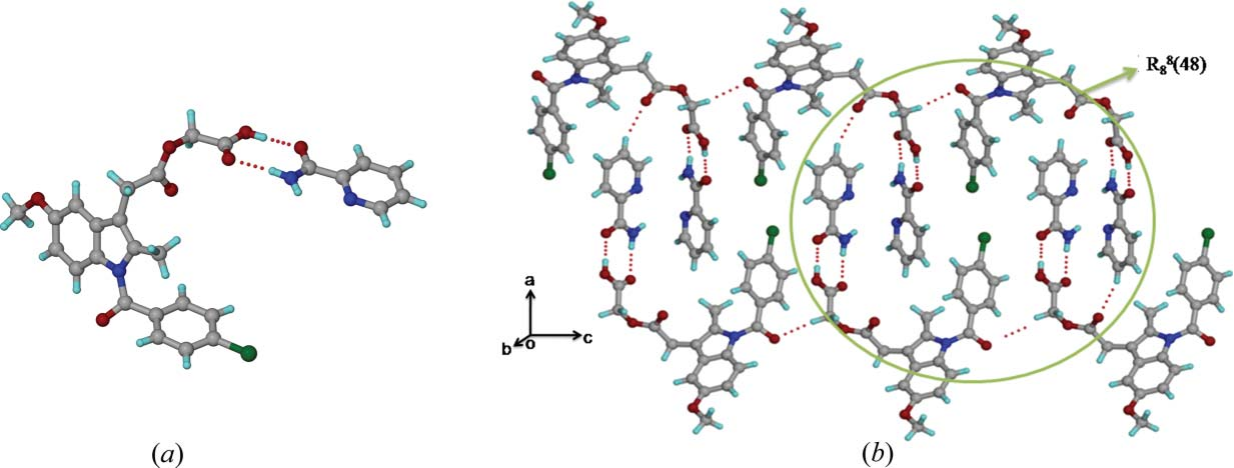
(*a*) Acid⋯amide heterosynthon in the ACM–PAM cocrystal. (*b*) N—H⋯O and auxiliary C—H⋯O interactions in the extended molecular arrangement.

The crystal structure of the ACM–PAM cocrystal (*P*2_1_, *Z* = 2) contains the acid⋯amide heterosynthon (N2—H2*A*⋯O5, O4—H4⋯O7; Table 2 ▶), comprising an *R*_2_^2^(8) ring motif (Fig. 2 ▶ *a*). Unlike ACM–INA, the acid⋯pyridine heterosynthon is not present, due to intramolecular hydrogen bonding between the amide group *ortho* to the pyridine N atom of picolinamide. Similar to the ACM–INA structure, the amide and ester carbonyl groups participate in auxiliary C—H⋯O interactions. ACM molecules form a one-dimensional chain parallel to the *c*-axis through C—H⋯O interactions between the amide carbonyl and the activated CH_2_ donor adjacent to COOH. The phenyl C—H of PAM makes auxiliary C—H⋯O interactions with the ester carbonyl of ACM. Four ACM and two PAM molecules form an *R*_8_^8^(48) ring motif (Fig. 2 ▶ *b*).

### 3.3. Acemetacin–caprolactam cocrystal (ACM–CPR, 1:1)   

**Figure 3:**
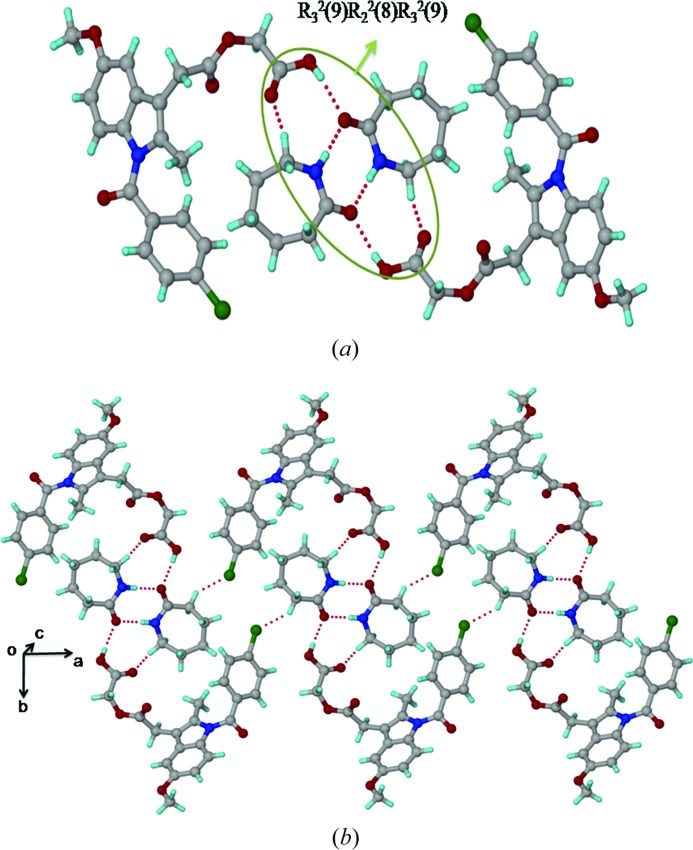
(*a*) Amide⋯amide homosynthon between CPR molecules, accompanied by O—H⋯O interactions between ACM and the coformer in the ACM–CPR cocrystal structure. (*b*) Tetramer units extend *via* auxiliary C—H⋯Cl interactions.

The structure of ACM–CPR (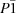, *Z* = 2) has one ACM and one CPR molecule in the asymmetric unit. CPR molecules form a centrosymmetric carboxamide dimer (N2—H2*A*⋯O7; Table 2 ▶) along with O—H⋯O (O4—H4⋯O7; Table 2 ▶) and C—H⋯O interactions between the carboxylic acid and CPR, to make tetramer units of the three-point synthon, *R*_3_^2^(9) *R*_2_^2^(8) *R*_3_^2^(9) (Fig. 3 ▶ *a*). These tetramer units are extended by C—H⋯Cl interactions (2.56 Å) between the acidic C—H donor (adjacent to C=O) of CPR and the Cl acceptor of ACM (Fig. 3 ▶ *b*).

### 3.4. Acemetacin–*para*-aminobenzoic acid adduct (ACM–PABA, 1:1)   

**Figure 4:**
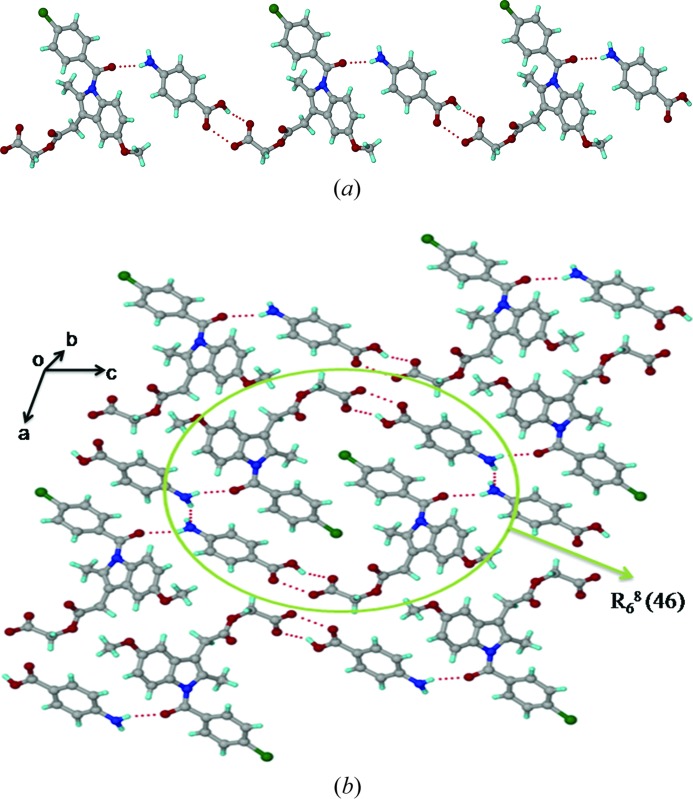
(*a*) N—H⋯O and O—H⋯O hydrogen bonds make a one-dimensional chain along the *c*-axis. (*b*) Extended packing in the ACM–PABA crystal structure, viewed down the *b*-axis. The acidic proton of ACM could not be located from the X-ray data and is not included in the figure.

The crystal structure of ACM–PABA (*P*2_1_, *Z* = 2) contains one ACM and one PABA molecule in the asymmetric unit. In this structure, it was not possible to locate the acidic H atom of the ACM molecule from the room-temperature single-crystal X-ray diffraction data. The bond distances in the carboxyl group of ACM [1.260 (13) and 1.248 (13) Å] are indicative of a carboxylate anion, but the O8⋯O6 interaction in the carboxyl(PABA)⋯carboxyl(ACM) heterosynthon offers the only viable site in the crystal structure to accommodate the acidic H atom. The amine group of PABA acts as a hydrogen-bond donor to the carbonyl of ACM (N2—H2*A*⋯O1; Table 2 ▶) and to the amine N atom of another PABA molecule (N2—H2*B*⋯N2; Table 2 ▶), and the intermolecular interactions indicate no possibility that the PABA-NH_2_ group could be further protonated. The mixed or difficult to assign proton state in ACM–PABA is consistent with a Δp*K*_a_ of 1.16 (Table 3 ▶). The carboxyl(PABA)⋯carboxyl(ACM) heterosynthon and N(PABA)—H⋯O(ACM) hydrogen bond (N2—H2*A*⋯O1; Table 2 ▶) make one-dimensional chains along the *c*-axis (Fig. 4 ▶ *a*), and screw-related PABA molecules form a chain of cooperative N2—H2*B*⋯N2 hydrogen bonds along the crystallographic *b*-axis (Fig. S1). Two ACM and four PABA molecules form a *R*_6_^8^(46) ring motif (Fig. 4 ▶ *b*).

### 3.5. Acemetacin–piperazine salt (ACM–PPZ, 1:0.5)   

**Figure 5:**
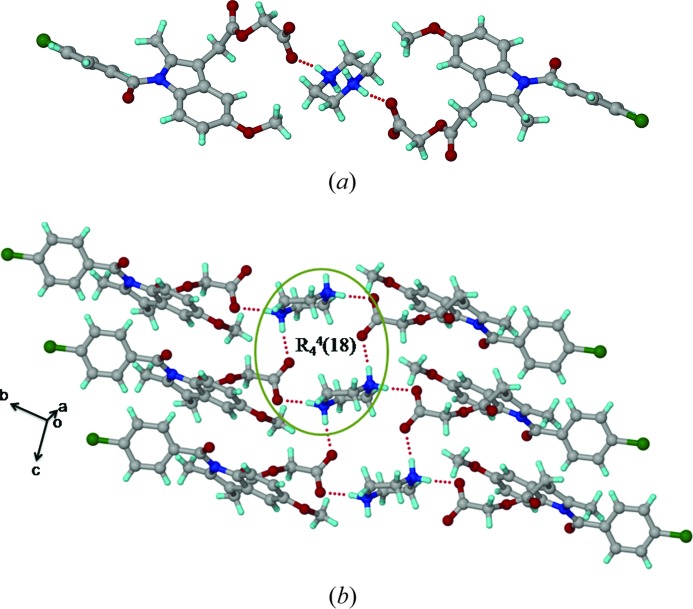
(*a*) Ionic N^+^—H⋯O^−^ interactions between two ACMs and one PPZ molecule. (*b*) Tetramer *R*_4_^4^(18) ring motif involving two ACM carboxylate and one piperazinium cation form a ladder-like structure.

The ACM–PPZ salt crystal structure (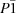, *Z* = 2) contains one acemetacin carboxylate anion and a piperazine dication, with the latter residing on the inversion centre. Two ACM carboxylate anions form N^+^—H⋯O^−^ ionic hydrogen bonds (N2—H2*A*⋯O5: Table 2 ▶) with a PPZ dication (Fig. 5 ▶ *a*). A tetramer ring motif *R*_4_^4^(18) is assembled *via* two ACM carboxylates and two PPZ cations, extended as a ladder motif along the *a*-axis (Fig. 5 ▶ *b*). There is a clear separation of hydrophobic (aromatic rings) and hydrophilic (carboxylate and piperazine cations) domains in this salt structure. The ester and amide carbonyls of ACM participate in auxiliary C—H⋯O interactions with the piperazine aliphatic CH_2_ and phenyl C—H adjacent to the Cl atoms. Salt formation of the piperazine dication matches with a large Δp*K*_a_ of 6.15.

### 3.6. Acemetacin–nicotinamide cocrystal (ACM–NAM, 1:1)   

We were unable to obtain good quality single crystals of ACM–NAM, even after several attempts.^2^ Attempts to solve the structure from PXRD data also were not successful. We were therefore unable to determine the structure of the ACM–NAM cocrystals as part of this study. The stoichiometry of ACM–NAM was confirmed as 1:1 by ^1^H NMR (Fig. S2). In a multi-component system containing a carboxylic acid, pyridine ring and carboxamide group, there is a high probability of acid⋯pyridine heterosynthons, followed by acid⋯amide or acid⋯acid (least probable) heterosynthons, following the strongest donor–strongest acceptor hydrogen-bonding rule (Etter, 1990 ▶; Aakeröy *et al.*, 2013 ▶). The ACM–INA cocrystals consist of the acid⋯pyridine synthon followed by a catemer motif of INA. However, in the ACM–PAM cocrystal, the pyridine N atom (being *ortho* to amide) does not participate in active hydrogen bonding, except an intramolecular hydrogen bond with the amide NH group.

### 3.7. Survey of the Cambridge Structural Database   

**Figure 6:**
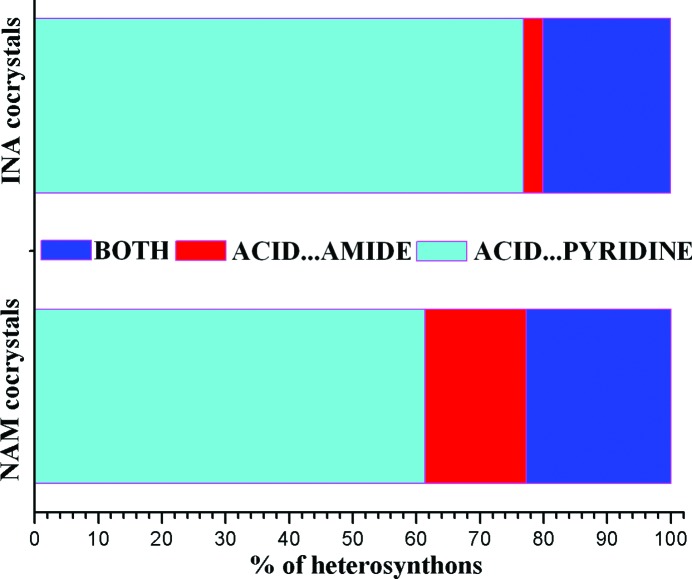
Comparison of heterosynthons in nicotinamide and isonicotinamide cocrystals with carboxylic acids present in the CSD.

The Cambridge Structural Database (CSD version 5.34, November 2012, May 2013 update; Allen, 2002 ▶) contains numerous cocrystals of the COOH functional group with nicotinamide (44) and isonicotinamide (65) (CSD refcodes listed in Table S1). There are no structural reports of picolinamide cocrystals with carboxylic acids. The acid⋯pyridine and acid⋯amide heterosynthons, or occasionally both, are observed in cocrystals of carboxylic acids with pyridine carboxamides (Table 4 ▶). The probability of occurrence of the acid⋯pyridine synthon in isonicotinamide cocrystals is higher than in nicotinamide (Fig. 6 ▶), perhaps because of a better solubility match of the less soluble isonicotinamide with the poorly soluble drug, whereas nicotinamide has excellent aqueous solubility (a hydrotrope). There are 17 carboxylic acids cocrystals with NAM and INA coformers (with three-dimensional coordinates determined) of different stoichiometry (Table S2). Among these, naproxen is the only drug having cocrystals with all three isomeric pyridine carboxamides. The X-ray crystal structures of naproxen with NAM (2:1) and INA (1:1) have been determined, whereas no three-dimensional coordinates are reported for the PAM adduct (Castro *et al.*, 2011 ▶; Ando *et al.*, 2012 ▶). The crystal structure of a PAM cocrystal with acemetacin with three-dimensional coordinates determined is reported for the first time in this paper, and it is sustained by the acid⋯amide supramolecular heterosynthon.

### 3.8. Structural similarity in the cocrystals   

**Figure 7:**
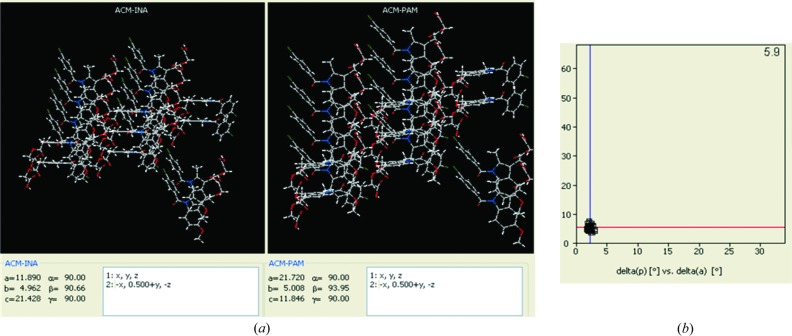
(*a*) Two-dimensional supramolecular construct of ACM–INA and ACM–PAM cocrystals, indicated by *XPac* analysis. (*b*) Inter-planar angular deviation (δ*p*, *x*-axis) *versus* angular deviation (δa, *y*-axis) (both in °) indicates a dissimilarity index of 5.9, which means that the two cocrystals form the same two-dimensional supramolecular construct.

The cocrystals ACM–INA and ACM–PAM adopt the monoclinic space group *P*2_1_ with very similar unit-cell parameters (Table 1 ▶), and they are three-dimensional isostructural (Cinčić *et al.*, 2008 ▶; Ebenezer *et al.*, 2011 ▶; Tothadi *et al.*, 2013 ▶; Figs. 1 ▶ *c* and 2 ▶ *b*). The isostructurality of ACM–INA and ACM–PAM (Fig. 7 ▶ *a*) was quantified by the *XPac* method (Gelbrich & Hursthouse, 2005 ▶, 2006 ▶; Gelbrich *et al.*, 2008 ▶). *XPac* is a program for comparing complete crystal structures based on the geometric conformations and positions of the molecules. Out of 14 near-neighbour molecules in a cluster, 10 molecules were matched in a two-dimensional supramolecular construct for ACM–INA and ACM–PAM. The dissimilarity index for the structures is 5.9 (Fig. 7 ▶ *b*), a number that is consistent with the kind of similarities observed in their PXRD line patterns (Fig. 9). Moreover, the ACM–CPR cocrystal (triclinic, 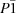) is three-dimensional isostructural with ACM–INA and ACM–PAM (monoclinic *P*2_1_). However, the dissimilarity index between ACM–PAM and ACM–CPR is slightly higher (9.1) than the other two pairs (Fig. S3). There is a difference in symmetry between the two structure types: ACM–INA and ACM–PAM adopt space group *P*2_1_, whereas ACM–CPR adopts space group 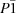. The isostructurality means that pseudo-inversion symmetry exists in the *P*2_1_ structures and pseudo-2_1_ symmetry exists in the 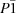 structures. Overall, the ACM molecules in all three structures adopt approximate *P*2_1_/*m* symmetry and similar molecular conformations.

### 3.9. Conformational analysis   

**Figure 8:**
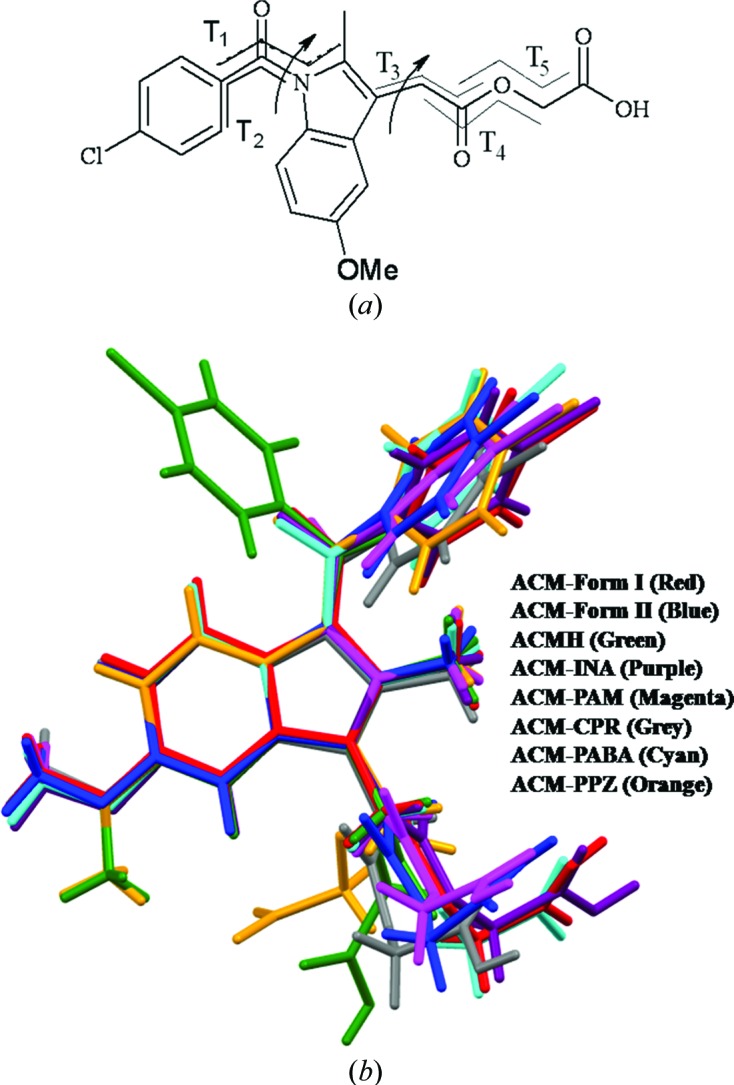
(*a*) Flexible torsion angles in ACM. (*b*) Molecular overlay of ACM polymorphs and its binary systems indicates torsional flexibility in the carboxamide and alkyl chain of the glycolic acid ester.

The indole ring in ACM is planar, but the glycolic acid ester side chain and *p*-Cl-benzoyl group have rotatable C—C bonds (Fig. 8 ▶ *a*). The torsional flexibility of the ACM molecule was noted in the two polymorphs and the known hydrate, and now in the cocrystal/salt structures reported here (Fig. 8 ▶ *b*). The orientation of the *p*-Cl-benzoyl group is different in ACM hydrate compared with the other crystal structures and also the orientation of the OMe group adopts a similar conformation in ACMH and ACM–PPZ. However, the glycolic acid part in ACM is flexible and shows variable conformations. The extended flexibility of the molecule is represented in the torsion angles τ_1_–τ_5_, as indicated Fig. 8 ▶ and Table 5 ▶.

### 3.10. Powder X-ray diffraction   

**Figure 9:**
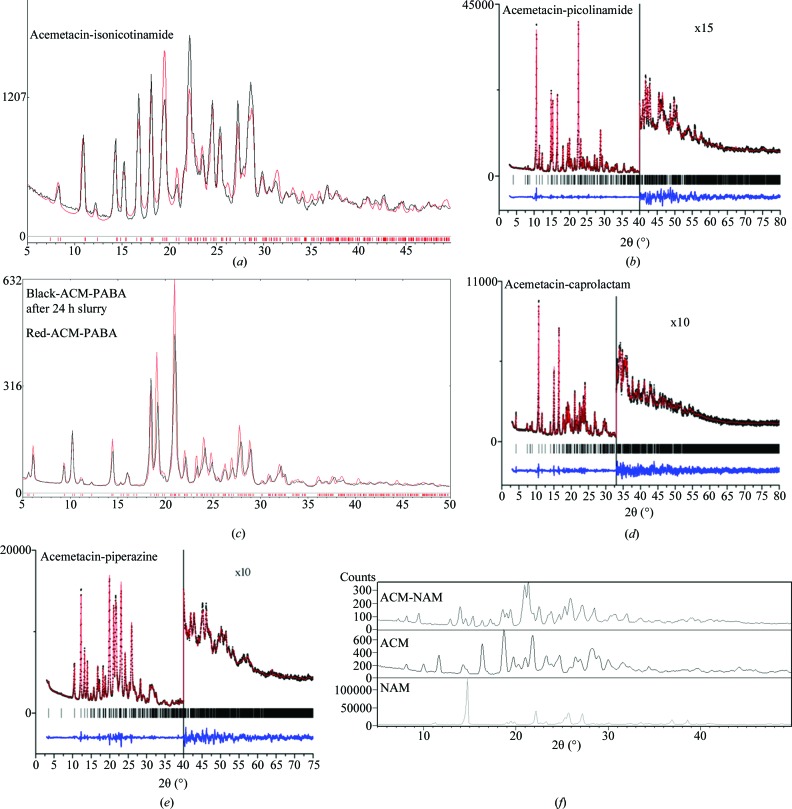
PXRD overlay (black trace) of (*a*) ACM–INA and (*c*) ACM–PABA with their calculated X-ray diffraction lines (red trace). Rietveld plots for (*b*) ACM–PAM, (*d*) ACM–CPR and (*e*) ACM–PPZ show the experimental (black dots), calculated lines (red) and difference (blue) plots. The vertical bars denote calculated positions of the diffraction peaks. (*f*) PXRD of ACM–NAM (1:1).

ACM readily transforms to a hydrate form during any kind of solvent-mediated crystallization. All of the cocrystals and salts were therefore prepared using dry solvents (preferably EtOAc), dry solvent-assisted grinding and melt crystallization (solvent free). The products were characterized to be free of the hydrate by PXRD fingerprint pattern matching (Fig. 9 ▶). The ACM–NAM cocrystal (whose X-ray crystal structure is still not determined) exhibited new diffraction lines compared with ACM and the coformer (Fig. 9 ▶ *f*). Even though the unit-cell parameters of ACM–INA and ACM–PAM structures are similar (Table 1 ▶), there are variations in their PXRD line patterns. The excellent overlay of the experimental and calculated X-ray diffraction patterns for the ACM–INA cocrystal and the ACM–PABA adduct (Figs. 9 ▶ *a* and *c*) indicate phase purity. The piperazine salt (1:0.5) contains half an equivalent excess of base in the solid phase (since a 1:1 stoichiometry was initially taken) and (1:0.5) salt was confirmed from the crystal structure and phase purity by DSC. The ACM–PPZ (1:1 and 1:0.5) salt melt samples provided similar PXRD and DSC profiles. There are a few weak PXRD lines that match with PPZ in the 1:1 crystallization product at high 2θ (see also discussion in the SS NMR section on the same point). After melt crystallization at 150°C, both the 1:1 and 1:0.5 products melt at 175°C and exhibit similar PXRD, IR and DSC.

### 3.11. FT–IR spectroscopy and DSC analysis   

**Figure 10:**
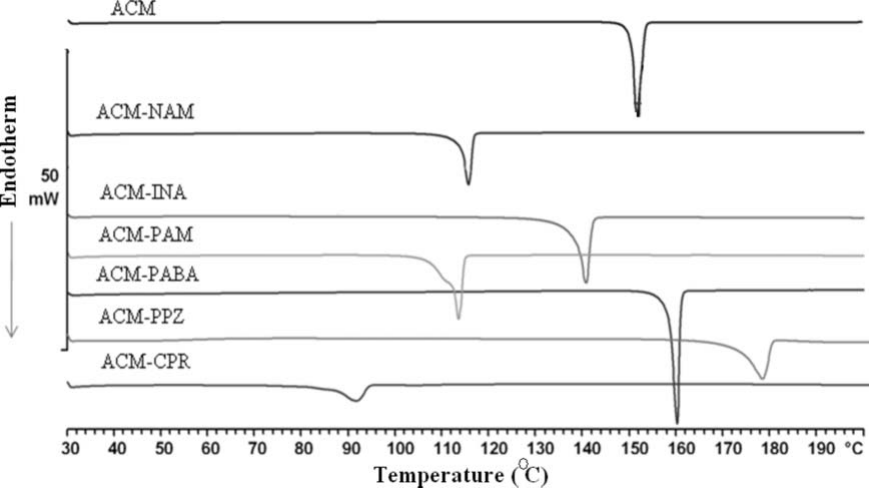
DSC endotherms of acemetacin and its multi-component molecular crystals.

Changes in the position and intensity of IR stretching and bending vibrations confirm the formation of new hydrogen bonds, notably for COOH and CONH_2_ functional groups. The distinction between cocrystal and salt (COOH *versus* COO^−^) was shown in the stretching vibrations (Fig. S4 and Table 6 ▶) of C=O and COOH group *versus* those for CO_2_^−^. The carbonyl vibration of the carboxylic acid in ACM–PABA shows a bathochromic shift from 1726 (ACM) to 1716 cm^−1^. The corresponding C=O peak shift for ACM–INA and ACM–PAM are 1727 and 1718 cm^−1^, respectively. On the basis of IR spectra, therefore, ACM–PABA may be defined as a cocrystal. Thermal analysis suggested a single homogeneous phase exhibiting a sharp melting point (Fig. 10 ▶ and Table 7 ▶). The broad melting endotherm for ACM–PAM may be due to dissociation of the cocrystal at the melting onset temperature, with PAM melting followed by the cocrystal phase transition.

### 3.12. Solid-state NMR spectroscopy   

**Figure 11:**
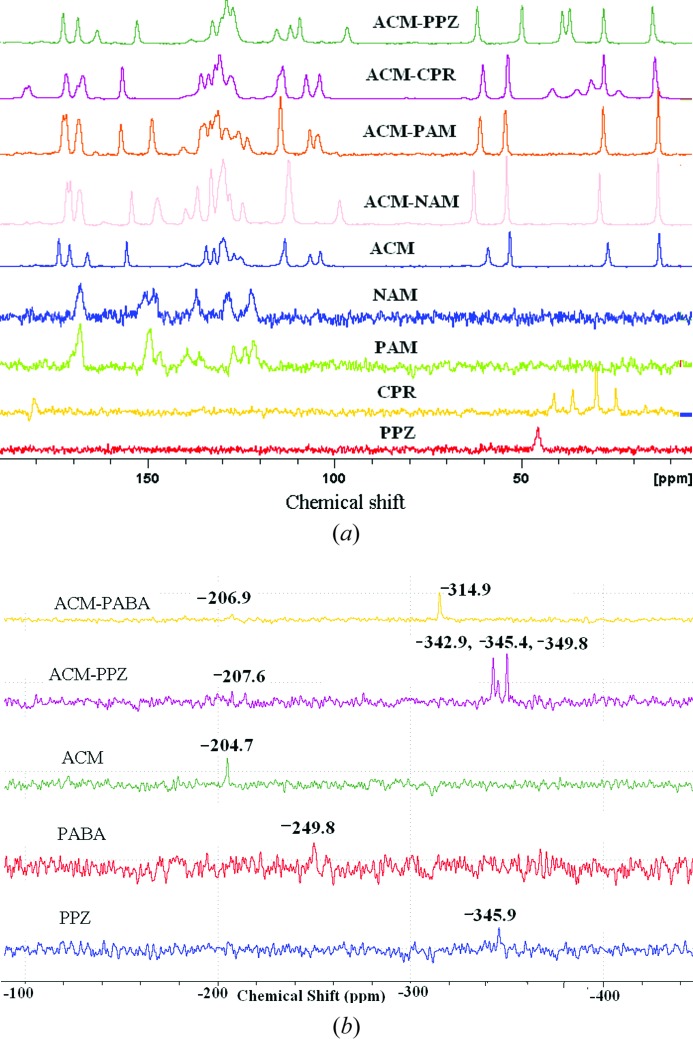
(*a*) ^13^C SS NMR and (*b*) ^15^N SS NMR spectra of acemetacin cocrystals and salts.

Solid-state NMR (Tishmack *et al.*, 2003 ▶; Vogt *et al.*, 2009 ▶; Widdifield *et al.*, 2013 ▶) is a generally reliable tool to differentiate between cocrystals and salts. Along with three kinds of carbonyl peaks in ACM (carboxylic acid, ester, carboxamide), the coformers too have C=O bond groups (except PPZ), which make a very difficult situation to assign the downfield peaks correctly due to overlapping C=O peaks in the ^13^C SS NMR spectra (see Fig. 11 ▶ *a* and δ values summarized in Table S3). Further, ^15^N SS NMR spectra were recorded to confirm the ionization state of the amine group in the coformers (Fig. 11 ▶ *b*). Peak intensities are very low in this case. The N-protonation of PPZ increases the magnetic shielding towards the upfield region from −345.9 to −349.8 p.p.m. for PPZ, thus confirming the ionic nature of the ACM–PPZ salt. The ^15^N spectrum of ACM–PPZ exhibits three peaks at −342.9, −345.4, −349.8 p.p.m., compared to one peak in free PPZ at −345.9 p.p.m. (the middle peak in the salt matches with that for free ACM). It is possible that there is partial proton migration along the cylindrical channel of N^+^—H⋯O^−^ hydrogen bonds (Fig. 5 ▶), and that there is some contribution from a PPZ monocation along with the dication observed in the crystal structure. Another possibility is that the grinding required to make the NMR sample and compression in the rotor could have induced proton migration. The large upfield shift of the amine N from −249.8 to −314.9 p.p.m. for PABA may be due to the possibility of cooperative N2—H2*B*⋯N2 hydrogen bonds (Fig. S1) and maximizing the hydrogen-bonding nature of PABA. ^15^N SS NMR indicates that ACM–PABA is a cocrystal instead of a salt.

### 3.13. Dissolution experiments and stability studies   

The solubility of ACM is 1.9 g L^−1^ in pH 7 buffer medium at 25°C (Castro *et al.*, 2001 ▶). The solubility of the drug is highly pH dependent and decreases to 20 mg L^−1^ at pH 5 (COO^−^/COOH equilibrium). The solubility of acemetacin hydrate (ACMH) is 1.6 g L^−1^ in pH 7 buffer medium. Our goal was to suppress hydration and thereby improve drug solubility through the formation of an ACM cocrystal/salt. Solubility experiments on all solid forms were carried out in pH 7 buffer medium because of higher solubility at neutral pH. The equilibrium solubility of acemetacin and its hydrate is 3.2 g L^−1^ and 1.6 g L^−1^ (37°C) at 24 h (Table 8 ▶), at which point the drug had largely transformed to the hydrate (according to PXRD analysis, Fig. S5). The ACM–PPZ salt and ACM–NAM cocrystal exhibited the highest solubility (26.8 and 22.0 g L^−1^). Solubility measurements are meaningful only if there is no phase transformation during the dissolution experiment. All the cocrystals transformed to ACMH within 24 h of the slurry experiment, whereas the salts were stable in the aqueous medium.

**Figure 12:**
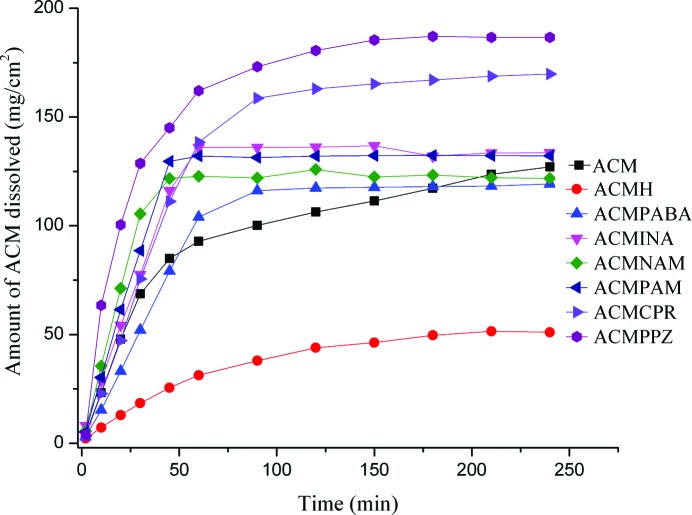
IDR measurements of acemetacin cocrystals/salts in pH 7 buffer medium.

For those solids which transform during the solubility run (*e.g.* to a polymorph or hydrate/ solvate), dissolution rates are a more useful guide to compare actual drug concentrations (Remenar *et al.*, 2003 ▶; Smith *et al.*, 2013 ▶). Intrinsic dissolution rate (IDR) measurements were carried out in pH 7 buffer medium. The ACM–PPZ salt and ACM–NAM cocrystal showed faster dissolution rates than the other solid forms, and more than 90% of ACM dissolved within 90 min (Fig. 12 ▶). Initially, the ACM–NAM and ACM–PAM cocrystals showed higher dissolution rates comparable to the ACM–PPZ salt, but after 45–50 min the concentration reached supersaturation. The ACM–CPR cocrystal showed a better dissolution profile at 90 min and was trailing below ACM–PPZ. For the ACM–PABA adduct and ACM–PPZ salt, the piperazine salt dissolves faster, following the solubility of the salt former (7 and 150 g L^−1^ for PABA and PPZ). All the cocrystals and salts were stable during the 4 h IDR experiment, confirmed by the absence of acemetacin hydrate peak at 1694 cm^−1^ in the IR spectrum. The ACM–PPZ salt and ACM–NAM cocrystal dissolve five times faster than acemetacin hydrate, but the ACM–PPZ salt is stable, while the ACM–NAM cocrystal transformed to acemetacin hydrate during the slurry experiment after 24 h.

**Figure 13:**
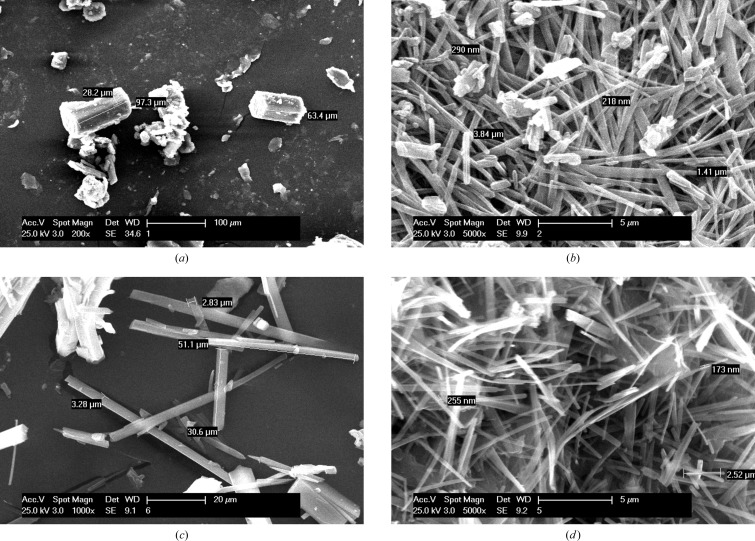
SEM images of acemetacin cocrystals and salts to show the crystal morphology.

The high solubility of ACM–PPZ is ascribed to the hydrophobic and hydrophilic domain separation in the salt structure (Fig. 5 ▶ *b*), which is absent in the other systems of this study. The morphology was examined in order to understand the dissolution and solubility behaviour of the cocrystals/salts. The block morphology of ACM–NAM cocrystals (see SEM images in Fig. 13 ▶ *a*), and the fact that NAM is a high solubility coformer, mean that this cocrystal has a high dissolution rate. The other cocrystals of needle morphology exhibit slower dissolution rates because of lesser contact surface area with the solvent. Generally, block crystals will dissolve faster than needle ones because of their higher surface area. Further, there is an inverse correlation between the dissolution rate and crystal density for binary systems (see density in Table 1 ▶ and solubility in Table 8 ▶). ACM–INA has the highest crystal density (1.413 g cm^−3^), packing efficiency and lowest dissolution rate, while the ACM–PPZ salt has a low crystal density and faster dissolution rate than the ACM–PABA adduct.

**Figure 14:**
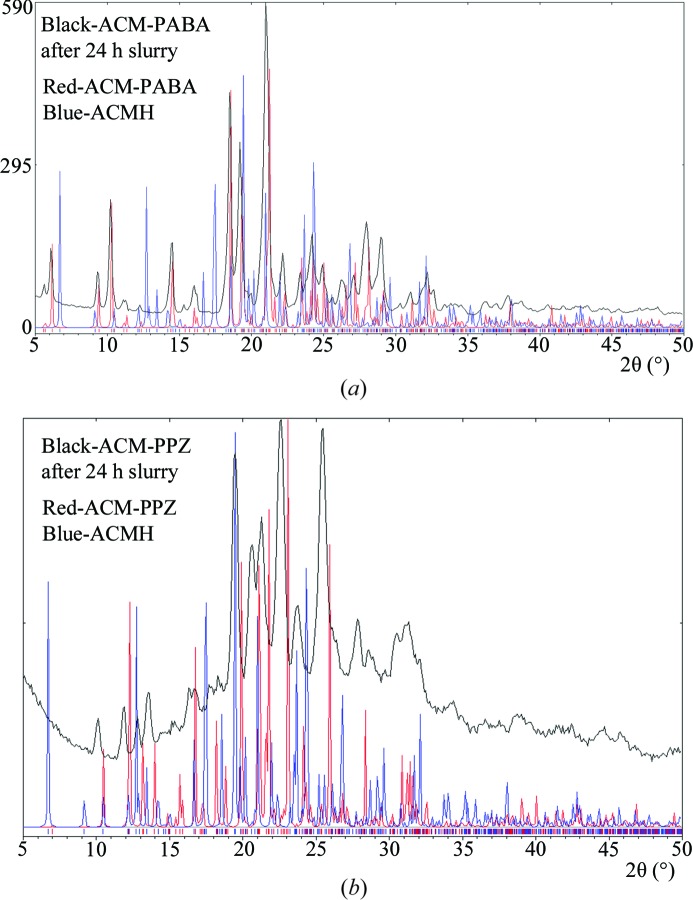
PXRD comparison (black trace) of (*a*) ACM–PABA and (*b*) ACM–PPZ salt after 24 h slurry with the calculated X-ray diffraction lines of the salt (red trace) and ACM hydrate (ACMH, blue). Both of these binary systems are relatively stable compared with the cocrystals, which transformed to ACMH in pH 7 buffer. There is a higher amorphous content in the recovered ACM–PPZ salt (halo + lines). The product stability in slurry medium was confirmed by FT–IR.

ACM and its cocrystals transformed to the hydrate in pH 7 buffer medium within 24 h of the slurry experiment (see PXRD in Fig. S5). The ACM–PAM cocrystal was somewhat stable at 24 h slurry. In comparison, the ACM–PABA adduct and ACM–PPZ salt are quite stable (Bolla & Nangia, 2012 ▶; Goud *et al.*, 2013 ▶; Perumalla *et al.*, 2013 ▶) at 24 h (Fig. 14 ▶). In summary, the ACM–PPZ salt provides a high solubility and relatively stable solid form for the BCS (Biopharmaceutics Classification System) class II drug acemetacin. A challenge for the next stage will be to crystallize this fast dissolving and stable ACM–PPZ salt in block or regular morphology crystals for ease of processing and manufacturing.

## 4. Conclusions   

Acemetacin prodrug reduces gastric damage compared with its parent drug indomethacin. The high tendency of acemetacin to transform to its monohydrate during crystallization and dissolution in aqueous medium is overcome by making a piperazinium salt. The binary phases were prepared in the salt screen by solidification of the melt phase followed by recrystallization from anhydrous solvents in dry conditions. The crystal structures of ACM–PAM, ACM–CPR cocrystals and the ACM–PPZ salt were obtained from high-resolution powder X-ray data. ACM–PAM and ACM–INA cocrystals showed similar unit-cell parameters and three-dimensional isostructural packing, and furthermore they are closely comparable with ACM–CPR. IR and SS NMR spectroscopy enabled the identification of cocrystals and salts based on the shift in resonance values. The ACM–PPZ salt exhibited the highest dissolution rate and superior stability in the aqueous medium. The ACM–NAM cocrystal has a comparably good dissolution rate but transformed to ACM hydrate after 24 h. This study has identified the ACM–PPZ salt as a high solubility and good stability form for an improved oral formulation of acemetacin.

## Supplementary Material

Crystal structure: contains datablock(s) global, ACM-CPR, ACM-INA, ACM-PABA, ACM-PAM, ACM-PPZ. DOI: 10.1107/S2052252514004229/bi5031sup1.cif

Structure factors: contains datablock(s) ACM-INA. DOI: 10.1107/S2052252514004229/bi5031ACM-INAsup2.hkl

Structure factors: contains datablock(s) ACM-PABA. DOI: 10.1107/S2052252514004229/bi5031ACM-PABAsup3.hkl

Rietveld powder data: contains datablock(s) ACM-CPR. DOI: 10.1107/S2052252514004229/bi5031ACM-CPRsup4.rtv

Rietveld powder data: contains datablock(s) ACM-PAM. DOI: 10.1107/S2052252514004229/bi5031ACM-PAMsup5.rtv

Rietveld powder data: contains datablock(s) ACM-PAM. DOI: 10.1107/S2052252514004229/bi5031ACM-PPZsup6.rtv

CIF: DFT-D optimized structures (unit-cell-constrained). DOI: 10.1107/S2052252514004229/bi5031sup7.txt

CIF: DFT-D optimized structures (unit-cell-free). DOI: 10.1107/S2052252514004229/bi5031sup8.txt

Details of CSD searches, additional NMR, FT-IR, PXRD data. DOI: 10.1107/S2052252514004229/bi5031sup9.pdf

CCDC references: 971218, 971219, 971220, 971221, 971222

## Figures and Tables

**Table 1:** Crystallographic details for the ACM cocrystals and salts

	ACMINA	ACMPAM	ACMCPR	ACMPABA	ACMPPZ
CCDC No.	971219	971221	971218	971220	971222
Crystal data					
Chemical formula	C_21_H_18_ClNO_6_ C_6_H_6_N_2_O	C_21_H_18_ClNO_6_ C_6_H_6_N_2_O	C_21_H_18_ClNO_6_ C_6_H_11_NO	C_21_H_18_ClNO_6_ C_7_H_7_NO_2_	(C_21_H_17_ClNO_6_ )_2_ C_4_H_12_N_2_^2+^
*M*_r_	537.94	537.94	528.97	552.96	917.76
Crystal system, space group	Monoclinic, *P*2_1_	Monoclinic, *P*2_1_	Triclinic, 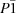	Monoclinic, *P*2_1_	Triclinic, 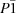
*T* (K)	298(2)	298(2)	298(2)	298(2)	298(2)
*a* ()	11.8900(8)	21.7202(15)	11.9406(16)	16.959(6)	7.3994(15)
*b* ()	4.9621(3)	5.0077(14)	21.3081(19)	4.7993(15)	25.6703(19)
*c* ()	21.4281(14)	11.8457(17)	5.1030(14)	17.285(5)	5.8254(17)
, , ()	90, 90.663(6), 90	90, 93.954(13), 90	92.373(15), 93.003(16), 85.308(17)	90, 113.55(4), 90	90.162(17), 98.598(16), 98.315(19)
*V* (^3^)	1264.16(14)	1285.4(4)	1291.3(4)	1289.7(7)	1082.2(4)
*Z*	2	2	2	2	1
_calc_ (gcm^3^)	1.413	1.390	1.360	1.424	1.408
(mm^1^)	0.204	1.763	1.728	0.204	1.939
Radiation	Mo *K*	Cu *K*	Cu *K*	Mo *K*	Cu *K*
range ()	2.8526.37	1.5040.00	1.5040.00	2.6224.71	1.5040.00
Specimen shape, size (mm)	0.28 0.16 0.12	Flat sheet, 15 1	Flat sheet, 15 1	0.24 0.12 0.12	Flat sheet, 15 1
					
Data collection					
No. of measured, independent and observed reflections	5380, 4397, 2600			4680, 3522, 1521	
*R*_int_	0.027			0.064	
values ()	_max_ = 26.4, _min_ = 2.9	2_min_ = 3.001, 2_max_ = 80.011, 2_step_ = 0.017	2_min_ = 3.000, 2_max_ = 80.000, 2_step_ = 0.008	_max_ = 24.7, _min_ = 2.6	2_min_ = 3.003, 2_max_ = 75.015, 2_step_ = 0.017
Distance from source to specimen (mm)	0.625			0.588	
					
Refinement					
*R* factors, goodness of fit	*R*[*F* ^2^ > 2(*F* ^2^)] = 0.068, *wR*(*F* ^2^) = 0.097, *S* = 1.04	*R*_p_ = 0.031, *R*_wp_ = 0.047, *R*_exp_ = 0.023, ^2^ = 4.005	*R*_p_ = 0.043, *R*_wp_ = 0.050, *R*_exp_ = 0.043, ^2^ = 1.381	*R*[*F* ^2^ > 2(*F* ^2^)] = 0.088, *wR*(*F* ^2^) = 0.144, *S* = 1.00	*R*_p_ = 0.029, *R*_wp_ = 0.038, *R*_exp_ = 0.023, ^2^ = 2.697
No. of reflections/data points	4397	4531	9626	3522	4237
No. of parameters	357	185	183	366	175
No. of restraints	3	125	123	4	107
_max_, _min_ (e ^3^)	0.19, 0.19			0.25, 0.26	
Flack parameter	0.03(11)			0.2(3)	

**Table 2:** Hydrogen-bond geometry (, ) for the crystal structures H-atom positions are normalized to average neutron-derived distances: CH = 1.089, NH = 1.015, OH = 0.993 (Allen Bruno, 2010 ▶).

	H*A*	*D* *A*	*D*H*A*	Symmetry code
ACMINA (1:1)				
N2H2*A*O3	1.97	2.977(3)	179	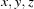
N2H2*B*O7	1.92	2.889(3)	161	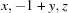
O6H6*A*N3	1.65	2.636(3)	177	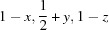
C2H2O6	2.29	3.369(3)	174	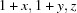
C12H12O1	2.32	2.892(2)	111	Intramolecular
C17H17*A*O3	2.46	3.483(3)	156	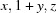
C20H20*B*O1	2.42	3.471(3)	164	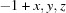
				
ACMPAM (1:1)				
N2H2*A*O5	1.91	2.866(4)	156	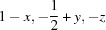
N2H2*B*N3	2.32	2.722(4)	102	Intramolecular
O4H4O7	1.55	2.518(4)	167	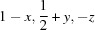
C2H2O4	2.27	3.333(4)	166	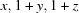
C20H20*A*O1	2.49	3.507(4)	156	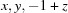
C26H26O2	2.42	3.455(4)	159	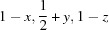
				
ACMCPR (1:1)				
N2H2*A*O7	1.97	2.955(4)	165	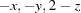
O4H4O7	1.90	2.665(4)	133	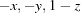
C18H18*B*O6	2.18	3.094(5)	140	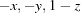
C23H23*A*Cl1	2.56	3.401(5)	134	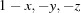
C27H27*B*O5	2.08	2.972(4)	138	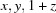
				
ACMPABA (1:1)				
N2H2*A*O1	2.02	2.965(4)	156	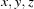
N2H2*B*N2	2.24	3.215(5)	163	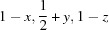
O7H7*A*O5	1.66	2.632(4)	168	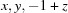
C12H12O1	2.35	2.888(6)	109	Intramolecular
C15H15O2	2.40	3.458(6)	166	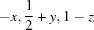
C17H17*A*O3	2.42	3.493(6)	172	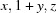
C18H18*A*O3	2.38	3.437(6)	164	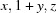
C25H25O1	2.33	3.267(6)	144	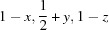
				
ACMPPZ (1:0.5)				
N2H2*A*O4	2.51	3.226(4)	128	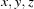
N2H2*A*O5	1.63	2.619(3)	166	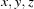
N2H2*B*O4	1.80	2.798(3)	172	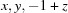
C2H2O1	2.35	3.202(4)	134	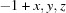
C13H13O1	2.13	2.739(4)	113	Intramolecular
C17H17*B*O3	2.20	3.098(4)	139	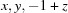
C20H20*A*O4	2.34	3.055(4)	122	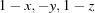
C22H22*A*O5	2.33	3.362(4)	158	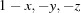
C22H22*B*O2	2.34	3.020(4)	119	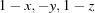

**Table 3:** Coformers used to make cocrystal/salts with ACM and corresponding p*K*_a_ values p*K*_a_ values were calculated in water using a SPARC p*K*_a_ calculator, http://archemcalc.com/sparc/test/login.cfm (accessed 16 February 2014).

	p*K*_a_	p*K*_a_	Cocrystal/salt
ACM	3.57		
NAM	3.31	0.26	Cocrystal
INA	4.17	0.6	Cocrystal
PAM	2.95	0.62	Cocrystal
CPR	0.90	2.67	Cocrystal
PABA	2.41	1.16	Cocrystal
PPZ	9.72	6.15	Salt

**Table 4:** Summary statistics of acidpyridineamide heterosynthons in cocrystals in the CSD

Heterosynthon	NAM	INA	PAM
Acidpyridine	27	50	0
Acidamide	7	2	0
Both	10	13	0
Total	44	65	0

**Table 5:** Torsion angle () variation in ACM crystal structures (see Fig. 8 ▶)

	_1_	_2_	_3_	_4_	_5_
ACM form (I)	27.9	53.3	175.2	179.9	78.4
ACM form (II)	35.7	38.0	172.7	172.3	81.3
ACMH	154.2	48.6	7.8	180.2	68.5
ACM-INA	29.8	49.4	179.8	178.0	79.9
ACMPAM	23.4	51.9	179.9	160.6	96.4
ACMCPR	35.3	40.1	172.3	160.1	76.8
ACMPABA	43.6	40.6	179.5	179.8	75.7
ACMPPZ	18.2	36.4	46.9	148.8	149.9

**Table 6:** IR frequency (cm^1^) of the acemetacin cocrystals/salts

	NH stretch	CO stretch	CO stretch	CO stretch (coformer)
ACM		1751.2, 1726.5, 1665.8	1229.5	
ACMNAM	3403.4, 3304.9, 3216.1	1735.2, 1669.0	1226.2	1698.3, 1681.0
ACMINA	3395.1, 3311.3, 3264.5, 3208.7	1727.0, 1672.6	1227.8, 1213.2	1677.2
ACMPAM	3445.6, 3309.4	1741.4, 1717.9, 1667.8	1232.6	1683.3
ACMCPR	3445.4 (broad)	1738.3, 1668.9	1234.1	1636.0 (broad)
ACMPABA	3464.7, 3402.2, 3332.9, 3227.2	1746.4, 1716.1, 1643.7	1234.5, 1216.4	1687.3, 1662.7
ACMPPZ	3423.0 (broad)	1721.9, 1679.0	1219.6	**

**Table 7:** Melting point (C) of acemetacin cocrystal/salts

	M.p.	M.p. of coformer
ACM	150.6151.3	
ACMNAM	115.3116.7	128131
ACMINA	138.4140.4	158159
ACMPAM	111.6113.1	109110
ACMCPR	92.893.9	6870
ACMPABA	158.2159.2	187189
ACMPPZ	173.2178.1	106108

**Table 8:** Dissolution of acemetacin cocrystals/salts in pH 7 buffer medium at 37C

	Absorption coefficient (, mM^1^cm^1^)	Solubility at 24h (gL^1^)†	IDR (mgcm^2^min^1^)†	Solubility (gL^1^) of the coformer	Residue after 4h in IDR	Residue after 24h slurry
ACM	7.71	3.2 (2.0)	2.118 (3.4)		ACMH	ACMH
ACMH	6.49	1.6	0.618		ACMH	ACMH
ACMNAM	5.93	22.0 (13.7)	3.223 (5.2)	800	ACMNAM	ACMH
ACMINA	7.06	21.0 (13.1)	1.165 (1.8)	192	ACMINA	ACMH
ACMPAM	6.78	17.2 (10.7)	2.902 (4.7)	180	ACMPAM	ACMPAM, ACMH
ACMCPR	7.15	20.5 (12.8)	2.195 (3.5)	4560	ACMCPR	ACMH
ACMPABA	6.77	21.6 (13.5)	1.631 (2.6)	7	ACMPABA	ACMPABA
ACMPPZ	6.78	26.8 (16.7)	3.260 (5.3)	150	ACMPPZ	ACMPPZ

† Value in parenthesis is the enhancement multiple compared to the least soluble ACMH.

## Footnotes

1 During the review process, all five crystal structures were energy minimized using dispersion-corrected density functional theory (DFT-D) calculations. For ACM–PABA, the H atom that could not be located from the X-ray data was placed on atom O6. The minimized structures with constrained and unconstrained unit cells are included in the supporting information. In all cases, the deviations on minimization are small and consistent with correct crystal structures (Van de Streek & Neumann, 2010 ▶).

2 In the course of this work, we did obtain single crystals that were identified as a channel solvate of the ACM–NAM co-crystal. The X-ray data and resulting structure quality were relatively poor, and we are working further to characterize this system.
