# Supplementary material for: Acemetacin cocrystals and salts: structure solution from powder X-ray data and form selection of the piperazine salt
Source: IUCrJ. 2014 Feb 28;1(Pt 2):136–50. doi: 10.1107/S2052252514004229 (PMC4062091; doi:10.1107/S2052252514004229)
Supplement: Supplementary file 9 [file m-01-00136-sup9.pdf]

Supporting Information

Acemetacin Cocrystals and Salts:
Structure Solution from Powder X-ray Data and Form Selection of the Piperazine Salt

Palash Sanphui,[†] Geetha Bolla,[†] Ashwini Nangia,[†,]* and Vladimir V. Chernyshev,[#,‡,]*

[†]School of Chemistry, University of Hyderabad, Prof. C. R. Rao Road, Central University P.O., Hyderabad 500046, India. E-mail: ashwini.nangia@gmail.com

[#] Department of Chemistry, M.V. Lomonosov Moscow State University, 1-3 Leninskie Gory, Moscow 119991, Russian Federation,

[‡] A. N. Frumkin, Institute of Physical Chemistry and Electrochemistry RAS, 31 Leninsky prospect, Moscow 119071, Russian Federation. E-mail: vladimir@struct.chem.msu.ru

Table S1 CSD version 5.34, November 2012, May 2013 update.

**Refcodes of carboxylic acid cocrystals with nicotinamide (NAM): Total 44 hits.**						
Acid···pyridine heterosynthon: 27 hits (61.4%)						
ABULEQ	ABULIU	CUYXUQ	DUZPAQ	EXAQAW	EXAQEA	EXAQIE
EXAQOK	FIFLAI	IACNCA	JEMDIP	JILZOU	NUKXUN	NUKYOI
NUKZEZ	NUKZOJ	PAMXAE	PEQBES	SODDIZ	SODDOF	SOGLAC
SUTLUX	UCOTUC	YASGOQ	JILZOU01	EDAPOQ	PEKRUU	
Acid···amide heterosynthon: 7 hits (15.9%)						
MUPMOA	MUPNIV	MUPPAP	RUYHEZ	XAQQIQ	YASGOQ03	YASHIL
Both acid···pyridine and acid…amide heterosynthons: 10 hits (22.7%)						
NUKYAU	NUKYEY	NUKYIC	NUKYUO	NUKZAV	NUKZID	PAMXIM
XAQPUB	HEGGAD	NUKYAU01				

**Refcodes of carboxylic acid cocrystals with isonicotinamide (INA): Total 65 hits.**						
Acid···pyridine heterosynthon: 50 hits (76.9 %)						
SAXPAK	AJAKAY	AJAKEB	ASAXOH	ASAXUN	BUDWEC	CECGUK
HANHEL	JAWWAG	JAWWEK	LUNMEM	LUNMIQ	LUNMOW	LUNMUC
LUNNAJ	LUNNEN	LUNNIR	LUNNOX	LUNNUD	LUNPAL	LUNPEP
MELYEI	MUPNUH	OCEBUV	PAMQAX	PAMWUX	ROLFOO	ROLFUU
RONDAA	RONDEE	RONDII	ULAWAF	ULAWEJ	ULAWOT	ULAWUZ
ULAXAG	UMUYUX	UMUZAE	UYOSUX	UYOTAE	UYOTEI	UYOTIM
UYOTOS	UYOTUY	UYOVAG	UYOVEK	XAQQEM	ACOMOW	ACONEN
ACONIR						
Acid···amide heterosynthon: 2 hits (3%)						
MUPNER	VAKTOR					
Both acid···pyridine and acid···amide heterosynthons: 13 hits (20%)						
AJAKIF	BUDZUV	BUFBIP	BUFQAU	HANBOO	ISIJAW	ISIJEA
ISIJIE	LUNMAI	MOVTOH	ULAXEK	XAQPOV	ACOMUC	

**Refcodes of carboxylic acid cocrystals with picolinamide : Total 0 hits.**
No picolinamide: carboxylic acid cocrystals are reported in the CSD.

Table S2 Summary of recodes of carboxylic acids cocrystals with both NAM and INA.

Carboxylic acids	INA cocrystals	NAM cocrystals
Succinic acid	LUNNUD	DUZPAQ
Fumaric acid	LUNNOX	EDAPOQ
Ferulic acid[a]	MUPNER	MUPNIV
Gallic acid[a]	MUPNUH	MUPPAP
Malonic acid	ULAWEJ	NUKXUN
Glutaric acid	ULAXAG	NUKYEY
Adipic acid	ULAXEK	NUKYIC
Adipic acid	ULAWUZ	NUKYOI
Pimelic acid	ISIJEA	NUKYUO
Suberic acid	ISIJIE	NUKZEZ
Azelaic acid	ISIJAW	NUKZID
Pyridine-2,6-dicarboxylic acid[a]	MELYEI	PAMXAE
4-Hydroxybenzoic acid	VAKTOR	RUYHEZ
1-Hydroxybenzoic acid	XAQQEM	SODDOF
3-hydroxybenzoic acid	LUNMEM	XAQQIQ
Naproxen	PAMQAX	---[b]
Fisetin[a]	CCDC no. 938498	EtOH hemisolvate CCDC no. 938496

[a] Note indicates cocrystal hydrates.

[b] No refcode is available.

Table S3 ^{13}C SS-NMR chemical shift (δ, ppm) of all the binary systems of ACM and coformers.

	**Carbonyl**	**Ester**	**Amide**	**Aromatic**	**AliphaticCH$_2$**	**OMe**	**Me**
ACM	175.0	172.0	167.4	156.9,135.6,133.5, 131.0,128.3,126.4, 114.5,107.8,105.1	60.1, 28.0	54.2	14.2
ACM–INA	173.6	172.6	162.4, 178.9	148.4,140.7,139.5, 137.4,136.0,134.2, 131.2,127.4,119.9, 111.9,109.8	67.0, 33.6	59.6	18.7
ACM–NAM	171.5	170.6	168.0	154.2, 147.2, 139.7, 136.6, 132.9, 129.7, 127.9,124.5, 112.1, 98.5	62.6, 28.9	53.7	13.29
ACM–PAM	172.5,	171.2	168.3	157.0,148.8,140.1, 135.6,134.7,133.1, 131.7,128.8,126.5, 125.6,123.2,114.3, 106.5,104.2	60.8, 27.9	54.1	13.1
ACM–CPR	171.7	168.6	167.2, 181.7	135.6, 131.8,130.6, 127.7, 113.7, 107.4, 103.8	60.1, 27.7, 41.5, 34.9, 31.1, 23.8	53.5	14.0
ACM–PABA	172.5	170.0	167.8	154.9, 150.8,133.3, 132.7,131.7,129.9, 125.0,116.7,115.9, 113.4,112.4,104.6	59.9, 26.6	54.2	12.9
ACM–PPZ	174.7	170.7	165.5	154.9, 134.7, 130.9, 129.2,117.5,113.8, 111.3,98.6	63.7,29.4, 41.3,39.0	51.8	16.8
INA			172.0	150.6,149.3,139.8, 120.4			
PAM			168.0	149.3,146.5,139.3, 135.5,127.0,123.7, 121.5			
NAM			168.9	152.4,148.3,136.9, 129.4,122.3			
PPZ					45.3		
PABA	179.0			158.1,135.8,121.5, 116.8			
CPR			180.4		24.9,29.7, 36.1,41.0		

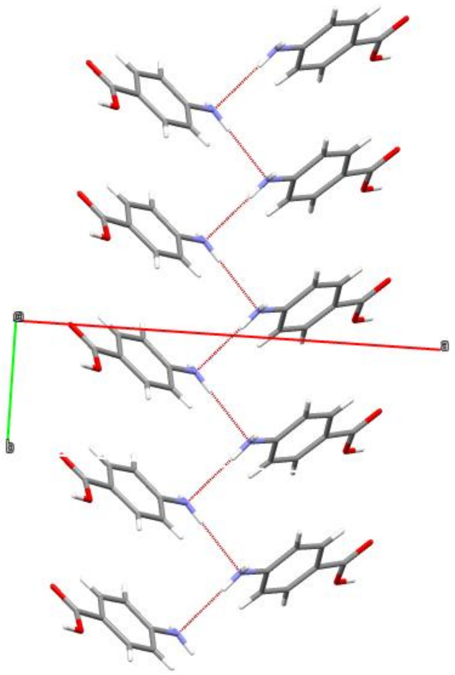

Figure S1 2_1 screw axis related PABA molecules form 1D chain via cooperative N–H···N hydrogen bonds along the *b*-axis.

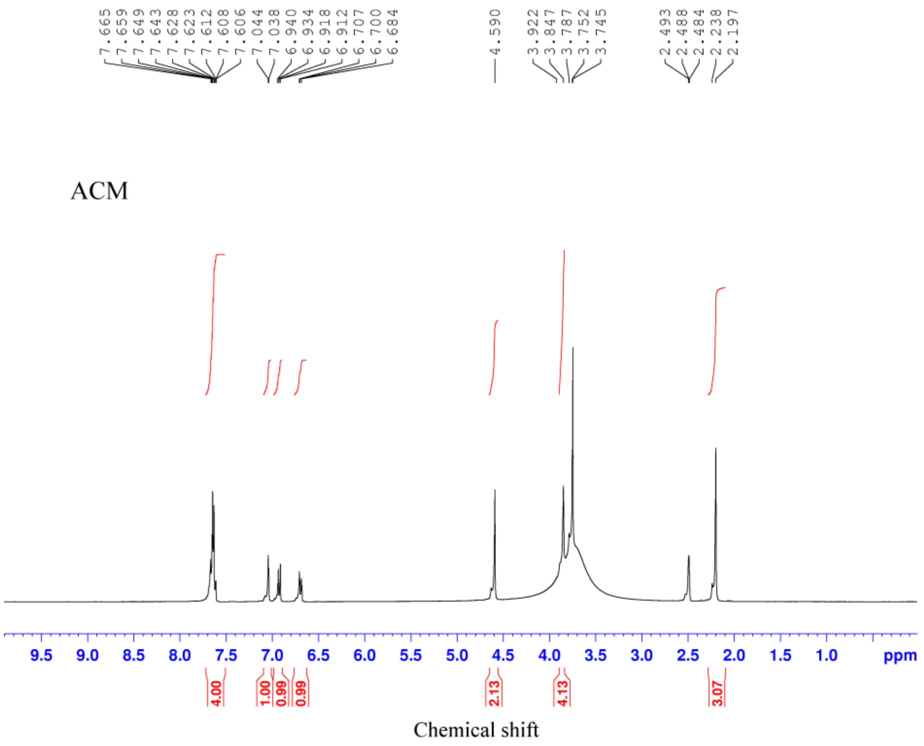

(a)

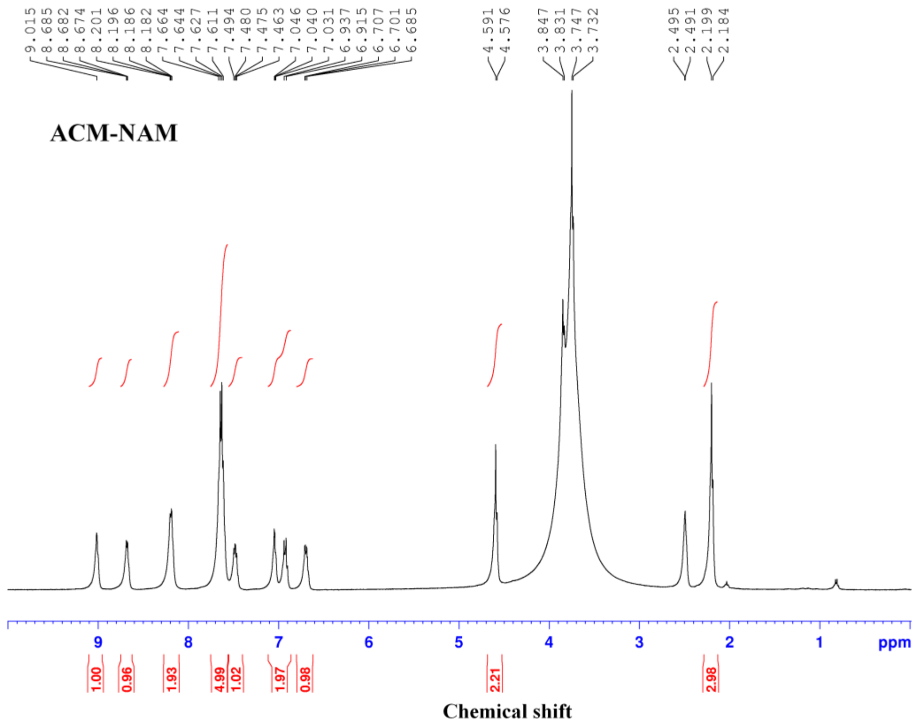

(b)

Figure S2 ^{1}H solution NMR of (a) ACM, (b) ACM–NAM in DMSO d^{6} solution suggests 1:1 stoichiometry between the ACM and NAM.

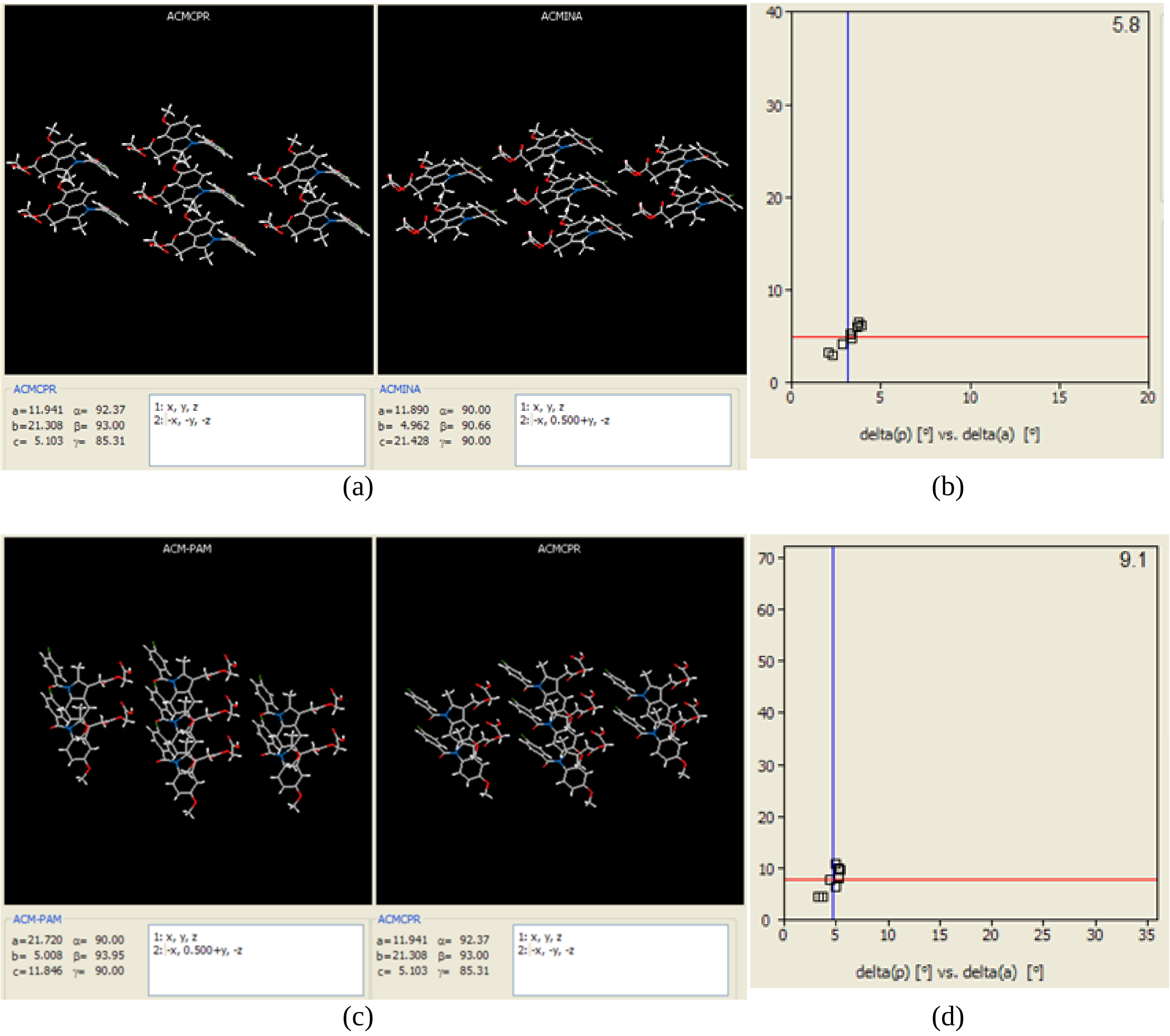

Figure S3 2D supramolecular construct of (a) ACM–CPR and ACM–INA and (c) ACM–PAM and ACM–CPR cocrystals was confirmed by XPac analysis. (b), (d) Inter-planar angular deviation (δp, x-axis) vs. angular deviation (δa, y-axis) plot (in º) indicates dissimilarity index of 5.9 and 9.1 which means cocrystals have the same 2D supramolecular construct.

(a)

(b)

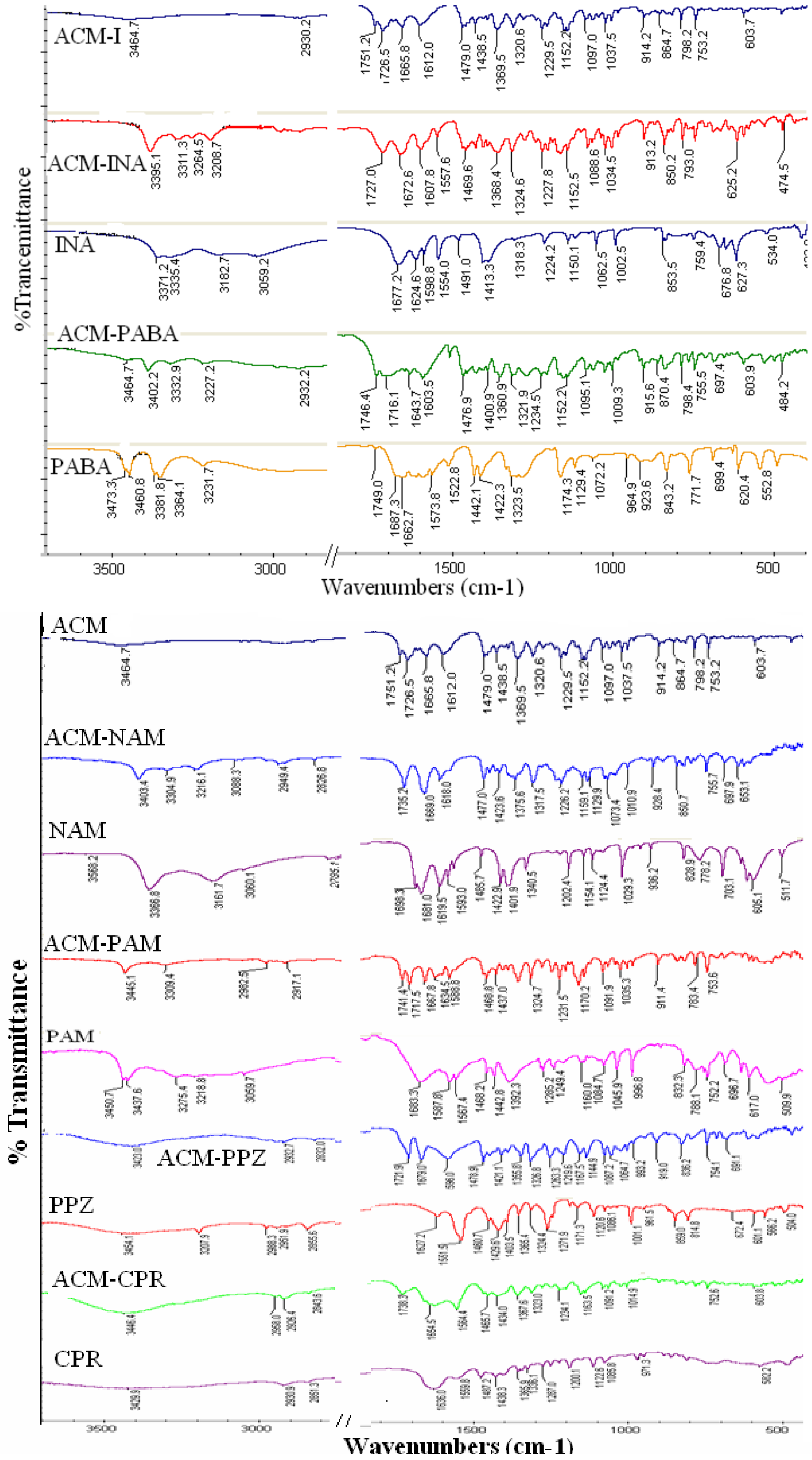

Figure S4 FT-IR spectra of ACM binary compounds indicate either cocrystal or salt formation.

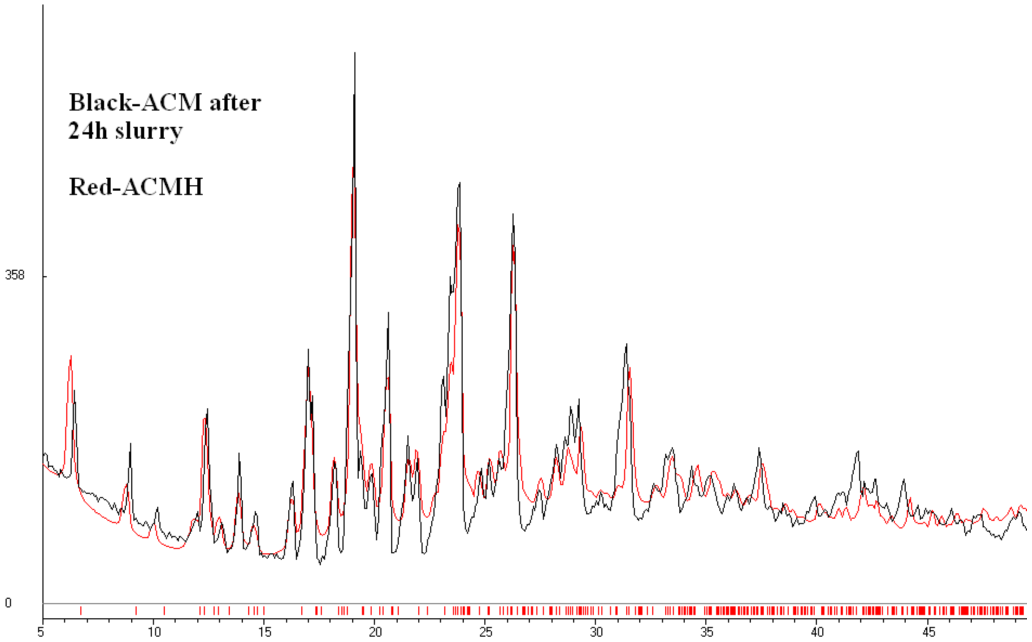
Black-ACM after
24h slurry
Red-ACMH
358
0
5
10
15
20
25
30
35
40
45

(a)

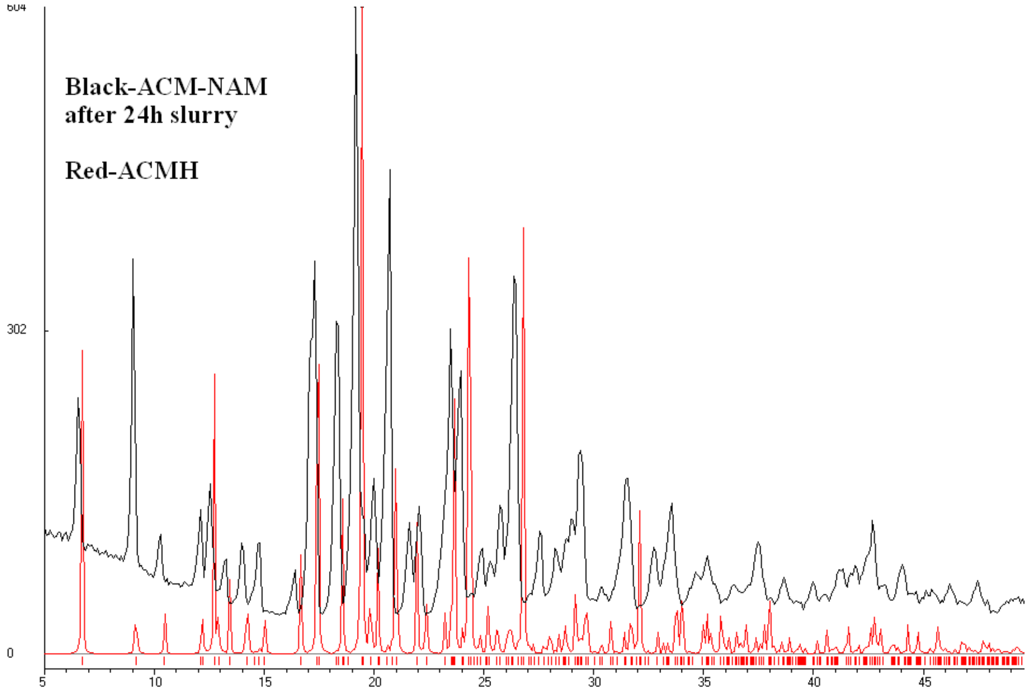
604
Black-ACM-NAM
after 24h slurry
Red-ACMH
302
0
5
10
15
20
25
30
35
40
45

(b)

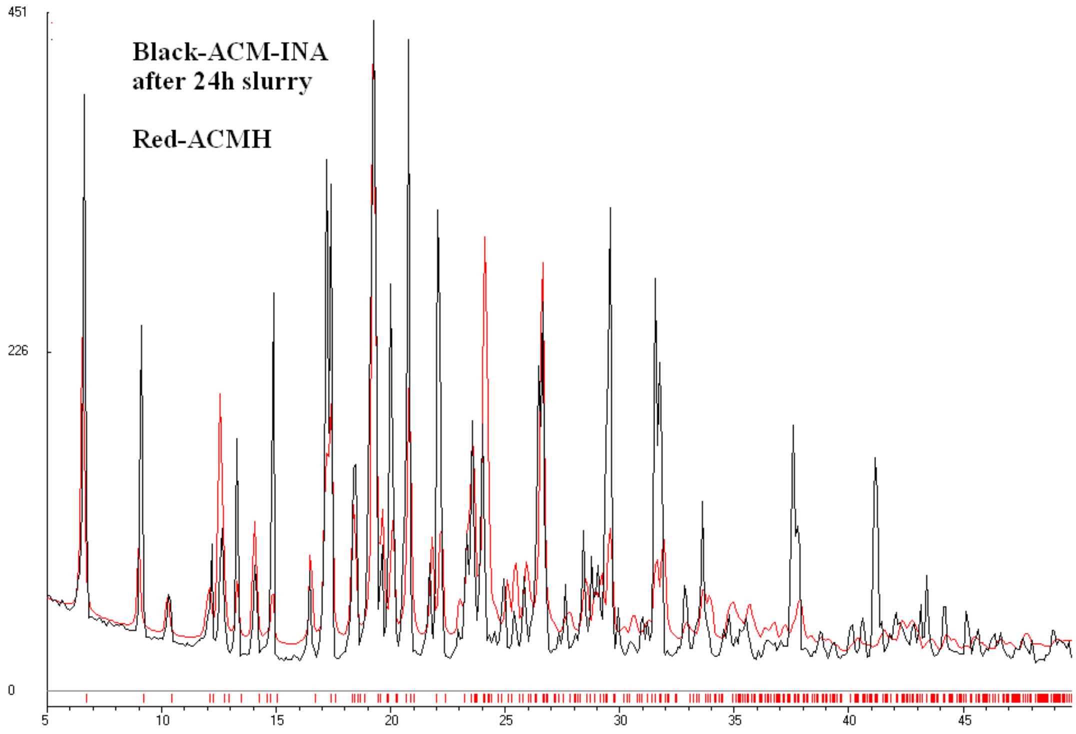
451
226
0
Black-ACM-INA
after 24h slurry
Red-ACMH
5
10
15
20
25
30
35
40
45

(c)

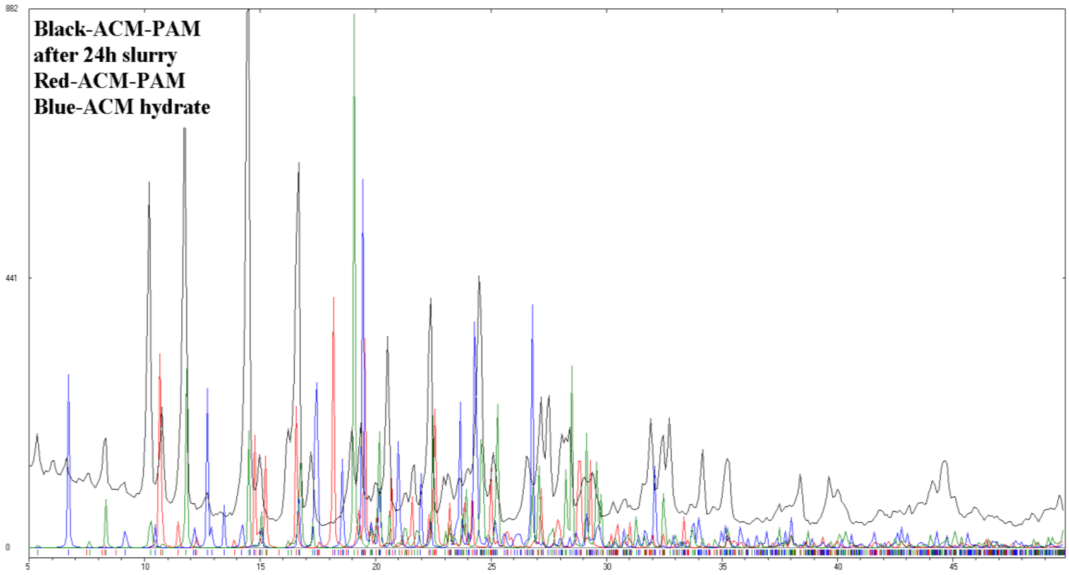
Black-ACM-PAM
after 24h slurry
Red-ACM-PAM
Blue-ACM hydrate

(d)

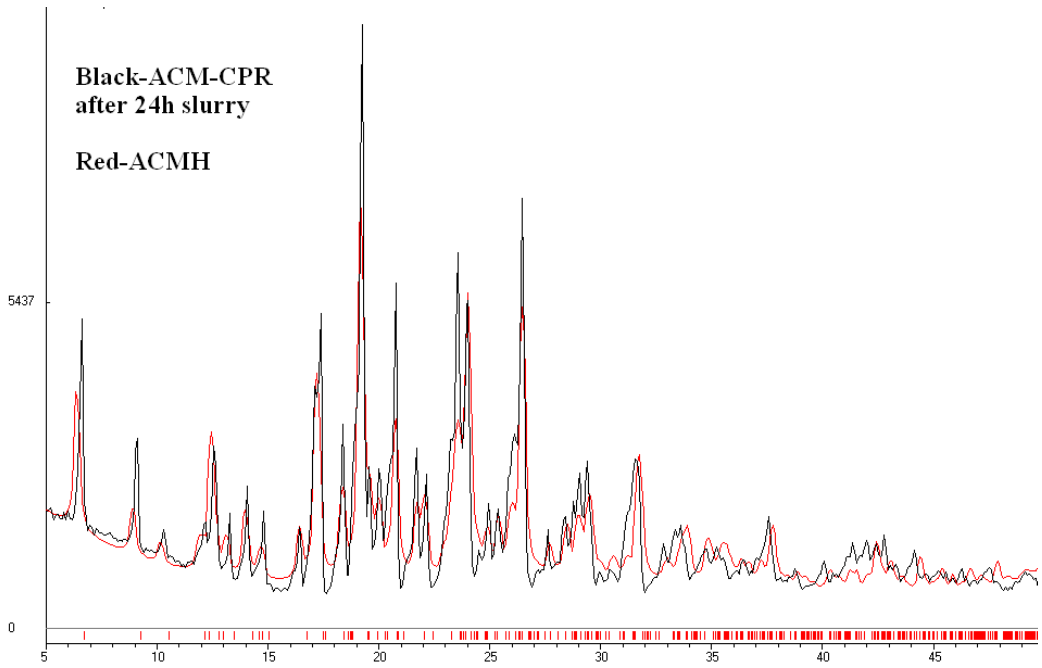

(e)

Figure S5 PXRD (black trace) of ACM (a) and its binary systems (b-e) after 24 h slurry experiments with the calculated X-ray diffraction lines (red trace) of acemetacin hydrate. ACM–PAM cocrystal showed that partial quantity of cocrystal still remained after 24 h slurry whereas the other cocrystals transformed completely to the hydrate in that period.
